# Development and evaluation of dried urine strip for genital chlamydia and gonorrhea testing

**DOI:** 10.1128/jcm.01618-25

**Published:** 2026-04-02

**Authors:** Suzanne Gibbons, Clarissa Klenke, Felicia Roy, Vanessa Schulz, Kimberly Ta, Sharon Simon, Jennifer Beirnes, Sabaparvin Shaikh, Ravinder Lidder, Christine Mesa, Shelley Peterson, Virginia Resh, Janet Fung, Jennifer Grant, Olivia Gemmell, Kevin Woodward, Paul Sandstrom, Alberto Severini, Irene Martin, John Kim, Aida Sivro

**Affiliations:** 1 National Microbiology Laboratory Branch, Public Health Agency of Canada, Winnipeg, Manitoba, Canada; 2 Department of Medical Microbiology and Infectious Diseases, Faculty of Health Sciences, University of Manitoba 423134, Winnipeg, Manitoba, Canada; 3 BCCDC Public Health Laboratory, BC Centre for Disease Control 113269, Vancouver, British Columbia, Canada; 4 HQ Toronto, Toronto, Ontario, Canada; 5 Department of Medicine, Division of Infectious Diseases, McMaster University 3710, Hamilton, Ontario, Canada; 6 Department Medical Microbiology, University of KwaZulu Natal, Durban, South Africa; 7 Centre for the AIDS Programme of Reserach in South Africa (CAPRISA), Durban, South Africa; Marquette University, Milwaukee, Wisconsin, USA

**Keywords:** chlamydia, gonorrhea, dried urine, filter paper, STI testing, genotyping, antimicrobial resistance

## Abstract

Here, we developed and evaluated a filter-paper urine collection method for chlamydia and gonorrhea testing. Dried urine strips (DUS) were developed to fit into the sample collection tube used for high throughput Hologic Aptima Combo 2 assay, minimizing processing post-sample collection. *Chlamydia trachomatis* (CT) and *Neisseria gonorrhoeae* (NG) bacterial stock dilutions were used to assess DUS performance and long-term stability at different temperature conditions. DUS diagnostic performance was evaluated against urine samples with Aptima Combo 2 Assay and an in-house RT-PCR method for CT and NG detection utilizing residual diagnostic urine specimens (*n* = 60 CT and NG negative, *n* = 50 CT-positive and *n* = 55 NG-positive, including 5 CT/NG dual-positive). Replacing the overnight strip drying step with use of additional desiccants was evaluated in order to reduce barriers to self-sample collection. We observed a high degree of diagnostic accuracy (>91% sensitivity and >98% specificity), compared to traditional urine samples across all evaluated DUS preparation and storage methods. DUS samples demonstrated high stability for prolonged periods at tested temperature storage conditions with some loss in sensitivity at lower bacterial loads. While drying of the sample was crucial to preserve sample integrity and improve recovery post-elution, placing the DUS directly into the sample storage bag with additional desiccants did not negatively impact diagnostic performance. DUS specimens were shown suitable for downstream CT genotyping and NG sequence typing. The developed DUS represents an accurate and stable sample collection method for CT and NG diagnostic testing and downstream pathogen characterization.

**IMPORTANCE:**

Innovative sampling methods that are convenient, accurate, and user-friendly are crucial to increase sexually transmitted infection (STI) testing uptake. This study establishes a novel dried urine strip sample collection method for chlamydia and gonorrhea testing and downstream molecular characterization. Results show no inferiority of dried urine strips compared to urine, with regard to diagnostic performance, when analyzed using molecular in-house and commercial assays for chlamydia and gonorrhea detection. A dried urine strip sample collection method for chlamydia and gonorrhea testing offers significant advantages in resource-limited or stigmatized settings; it enables self-collection, mail-in return, and long-term storage at ambient temperatures, making it ideal for reaching underserved populations. The broader public health impact is the expansion of STI screening access and reduction in STI transmission by offering a discreet, scalable alternative to traditional specimen collection.

## INTRODUCTION

Globally, more than one million new sexually transmitted infections (STIs) are acquired every day, driven partly by limited access to diagnostic testing in high-risk populations (1). Early testing, diagnosis, and treatment are key to reducing the rates of STIs, which, if left untreated, can lead to long-term negative health effects. While the majority of STIs are easily treated, treatment depends on access to testing, which is a barrier to STI management. People seeking STI screening and care face a number of challenges, including stigmatization and, in underserved regions, limited resources and out-of-pocket expenses. High-risk populations, including gay, bisexual, and other men who have sex with men, mobile populations, and adolescents, often do not have easy access to inclusive and adequate screening services. Furthermore, the asymptomatic nature of most STIs provides an additional barrier to screening and health service seeking behaviors.

Drying of biological samples is known to preserve genomic material by removing water, thereby inhibiting enzymatic degradation and preserving integrity and analytical viability (2, 3). The use of dried blood spot (DBS) sample collection has significantly reshaped screening for HIV, HCV, and syphilis (4–9). Drying of other sample types, including urine, was shown to be suitable for metabolome (10), and congenital cytomegalovirus (CMV) and schistosomiasis testing (11–13). In high-resource countries like Canada, systemic barriers to STI testing are being addressed through self-collection and point-of-care initiatives. DBS has proven effective in First Nations and Métis communities, underscoring the need for innovative testing strategies for other STIs not detectable in blood (8, 14).

Nucleic acid amplification testing (NAAT) of *Chlamydia trachomatis* (CT) and *Neisseria gonorrhoeae* (NG) is the diagnostic standard, and urine-based NAATs are widely used due to ease of sample collection and high analytical performance. However, the transport of liquid urine specimens requires leakproof packaging, adherence to specific biosafety and shipping requirements, and reliable transport systems. These logistical constraints can limit access to testing, particularly in remote or resource-limited settings where shipping biological liquids is challenging or costly. Although specimen transport media can maintain specimen stability for up to 30 days at room temperature, the underlying challenges associated with transporting liquid specimens remain.

Given these gaps, we sought to develop and evaluate a dried urine strips (DUS) as a sample collection method for genital chlamydia and gonorrhea testing, aiming to enhance care accessibility.

## MATERIALS AND METHODS

### DUS specifications

**Fig 1:**
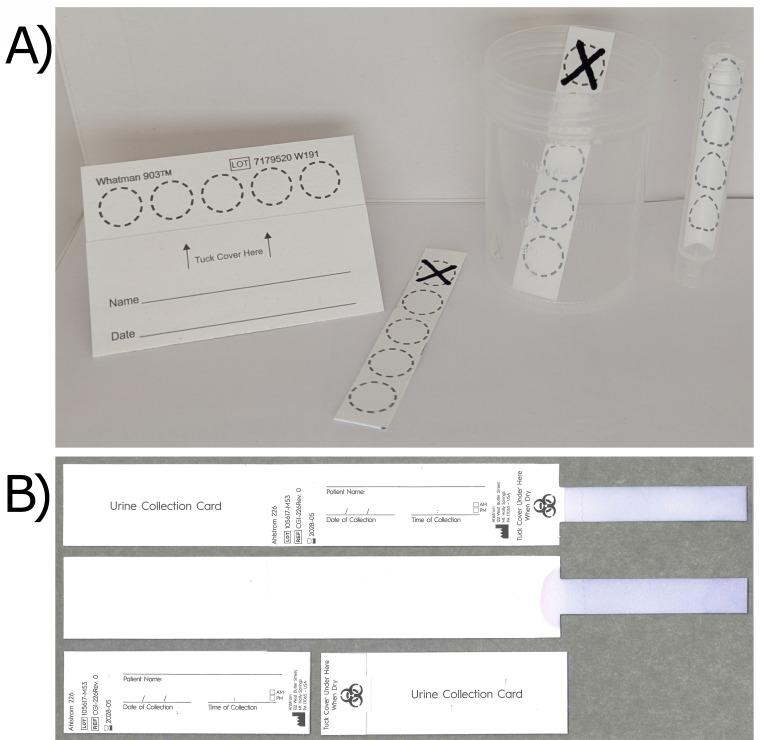
DUS specifications. (**A**) DUS specifications and design using Whatman 903 filter paper strip cut out from the WhatmanTM 903 Protein Saver card (approximately 13 mm × 68 mm). Item description from left to right: Whatman 903 DBS collection card; DUS strip cut from Whatman 903 cards used for 903 paper validation (X indicating excess portion of card maintained for user ease); Whatman 903 cut strip in a standard urine collection cup; Whatman 903 cut DUS strip inserted into Aptima tube with excess length removed. (**B**) DUS specifications and design using Ahlstrom grade 226 (A-226) filter paper. The card measures 105 mm folded and 290 mm in length when opened. The A-226 filter paper strip which is submerged in urine is 80 mm long × 15 mm wide and is perforated 10 mm from the tuck cover of the card to facilitate easy separation. Filter paper strips have been dyed blue for visualization purposes.

For initial evaluations, Whatman 903 Protein Saver cards were cut into strips that fit into the Aptima Specimen Collection tubes (Hologic, product FAB-18184). This allowed for immediate testing on the Hologic Panther, eliminating the need for strip transfer and reducing potential cross-contamination (Fig. 1A). The dimensions of the strip (approximately 13 mm × 68 mm) were optimized to maximize urine retention while preventing interference with instrument processes by ensuring adherence to the wall of the sample tube.

Based on the specifications provided by the National Microbiology Laboratory (NML) team, the Ahlstrom grade 226 (A-226) filter paper card (Fig. 1B) was produced by Ahlstrom Filtration LLC (USA). The card measures 105 mm folded and 290 mm in length when opened. The A-226 filter paper, which is submerged in urine, is 80 mm long × 15 mm wide and is perforated 10 mm from the tuck cover of the card to facilitate easy separation. The filter paper sample collection area absorbs ~800 µL of urine.

Due to the timing of the A-226 DUS card development, the initial laboratory evaluations using bacterial stock dilutions were performed using WhatmanTM 903 filter paper strips. Long-term stability was assessed using both WhatmanTM 903 and A-226 filter paper strips, and evaluations using clinical specimens were performed using the A-226 filter paper strips.

### DUS preparation

A urine collection cup/container, containing first void urine (~20 mL), was swirled to ensure the mixing of the sample. The filter paper strip was submerged in urine up to the perforations for approximately 5 s. The strip was removed from the cup and placed on a drying rack. The strips were sufficiently spaced to ensure no cross-contamination and left to dry at room temperature (22°C) overnight in a biological safety cabinet. The next morning, the strips were placed in the storage bag containing a clay desiccant and a humidity indicator card (Uline, products S-17703, S-5163, and S-8028, Fig. S1). Diagram of the procedure is shown in Fig. S2.

### DUS elution

Prior to testing, DUS was placed into an Aptima specimen collection tube (Hologic, product FAB-18184) using clean forceps. Strips were sized to fit snugly along the wall of the Aptima tube when capped with penetrable caps (Hologic, product #105668). Sample elution was done by adding 1.5 mL of Specimen Transport Medium (STM) (Hologic, PRD-04423) spiked with 4 μL of yeast tRNA (Invitrogen, product #AM7119) as a carrier to improve nucleic acid recovery. The filter strip absorbs ~800 µL of urine, resulting in a 1: 1.9 dilution post-elution. Samples were left at room temperature for 30 min on a nutating mixer at 30 rpm. Samples that were tested immediately were loaded directly onto the Hologic Panther system and tested with the Aptima Combo 2 Kit (Hologic, product PRD-05571/PRD-05576) without removal of the strip. For long-term sample storage, the filter paper was removed with tweezers and discarded in order to prevent paper degradation. The DUS elution volume was based on the need to have sufficient sample for duplicate testing for the Aptima Combo 2 Assay and/or downstream pathogen characterization. A diagram of the procedure is shown in Fig. S3.

### CT and NG testing

Primary CT and NG nucleic acid amplification testing (NAAT) was performed on the Panther Fusion instrument (Hologic, Cat. no. #303095, System Version 7.2.7.2), using the Aptima Combo 2 Assay (referred to as the Aptima test, Hologic, Cat. no. #PRD-05571/PRD-05576, Version: 5.3.5.1) following manufacturer’s instructions. For urine testing, STM (spiked with 4 μL of yeast tRNA) was added at a 1:1 ratio to urine (1:2 dilution). The Aptima Combo 2 Assay is a highly sensitive target amplification nucleic acid probe test that provides qualitative detection and differentiation of ribosomal RNA (rRNA) from CT and/or NG. This test is commonly used in Canadian provincial health laboratories for CT and NG detection in urine and other clinical specimens. The Aptima Combo 2 assay uses relative light units (RLU) measured in thousands (1,000×). The RLU is a measure of the chemiluminescence produced during the detection reaction. The instrument automatically interprets these RLU values as negative, equivocal, or positive based on predefined thresholds. RLU values that fall between the positive and negative threshold values are considered equivocal and require re-testing with a new specimen. The equivocal region for CT is 25 to <100 RLU (1,000×); for CT/NG dual infection, it is 85 to <250 RLU (1,000×), and for NG, it is 60 to <150 RLU (1,000×). While RLU values are not intended for precise quantification of bacterial load, they can be considered as semi-quantitative and used as a proxy for signal strength (amount of target rRNA). The presence of the filter paper strip in the DUS specimen tube did not interfere with the instrument processes.

### Nucleic acid extractions

Nucleic acid was extracted with the MagNA Pure 96 (MP96) System (Roche Diagnostics, Rotkreuz, Switzerland) using the DNA and Viral NA Large Volume kit (Cat. no. #06374891001), and eluted in 100 µL of 60 mM Tris-HCl (unless otherwise indicated).

### CT and NG real-time PCR (RT-PCR)

Nucleic acid extracted using the MagNA Pure 96 (MP96) system was tested with a previously described quadruplex RT-PCR assay for the detection of CT and differentiation of LGV and non-LGV serovars (15). The previously described NG RT-PCR assay was run as a single target reaction for the detection of the NG-specific *porA* gene (16). Cq (quantification cycle) values of the housekeeping *RNAseP* gene, CT cryptic plasmid, and NG *porA* gene were analyzed.

### Bacterial stocks

CT serovar G (ATCC VR-878) was cultured in HeLa 229 cells (ATCC CCL-2.1) as described previously (17). Briefly, infected cells were harvested in culture medium, centrifuged at 433 rcf for 5 min, and resuspended in sucrose-phosphate-glutamate (SPG) buffer. The cells were heat-inactivated at 65°C for 30 min and sonicated two times for 15 s at 40 MHz to release the bacteria. The suspension was centrifuged at 433 rcf for 5 min to remove cell debris, followed by concentration at 18,407 rcf for 10 min and resuspension in SPG buffer. NG strain F-18 (ATCC 49226) was grown on the Thayer-Martin agar. Colonies were picked and suspended in phosphate-buffered saline with 0.05% bovine serum albumin (PBS-BSA). NG dilutions for testing were pre-lysed using lysis buffer (Roche Diagnostics, Cat. no. #06374913001).

### CT and NG qPCR and bacterial stock dilutions

To quantify the prepared bacterial stocks, DNA was extracted using the Roche MP96 and quantified using modified CT RT-PCR and NG RT- PCR protocols (15, 18). Standard curves for qPCR in copies per mL were prepared for the NG *porA* gene from quantitative NG DNA (ATCC, Cat. no. #700825DQ) and an in-house plasmid construct containing an amplified region of the CT *pmpH* gene. Quantified bacterial stocks were diluted to an approximate concentration of 1 × 10^8^ copies/mL, from which 10-fold serial dilutions (1 × 10^7^ to 1 × 10^0^ copies/mL) were prepared and stored in 150 µL aliquots to avoid repeated freeze-thaw cycles.

### DUS assessment using prepared bacterial stocks

To assess target loss due to filter paper sample retention, bacterial stock dilutions for CT and NG were added to healthy donor urine (4 replicates/sample type). For urine, 20 µL of bacterial stock was added to 800 µL of urine (1 in 40 dilution). For DUS, 30 µL of bacterial stock was added to 800 µL of urine, and the resulting spiked urine was pipetted onto the filter paper strip. After overnight drying, DUS was eluted in 1,200 µL in order to maintain the same ratio of bacteria to final volume (1 in 40), while yielding sufficient eluate volume for testing. DNA from 400 µL of urine and DUS elutions was extracted and eluted in 50 µL. CT and NG qPCR was performed using 10 µL of extracted DNA. Bacterial concentration calculations are shown in Table S1.

DUS were further evaluated using prepared bacterial stock dilutions with the Aptima test. The outline of this procedure is shown in Fig. S4. For each bacterial concentration, spiked urine was prepared by adding 120 µL of bacterial stock to 4 mL of urine. From this, 1 mL of urine and three DUS cards were prepared. As negative controls, three 1 mL aliquots of urine and 12 DUS cards were prepared using unspiked urine. Each sample was tested in duplicate using the Aptima test, and the entire procedure was repeated five times. Raw data and associated calculations are shown in Tables S2 and S3.

### DUS stability testing

Stability testing was conducted using two separate bacterial dilutions: CT (urine sample concentrations of 3 × 10^3^ copies/mL and 3 × 10^2^ copies/mL); NG (urine sample concentrations of 3 × 10^3^ copies/mL and 30 copies/mL). DUS samples were prepared in triplicate for storage at room temperature (RT), 4°C, −20°C, and were processed on day 3, weeks 1, 2, 3, 4, and 6, and months 2, 3, 4, 5, 6, 9, and 12. Day 3, week 1, and week 2 time points have only one replicate due to bacterial stock volume limitations. DUS samples were prepared for storage at +30°C for the first 2 months and at −80°C for months 3 to 12. At each time point, the DUS were removed from the storage condition and eluted. The following elution samples were stored at −80°C until ready for batch testing and were tested once using the Aptima test.

### Clinical specimen collection and processing

Leftover clinical specimens from routine chlamydia and gonorrhea testing were obtained from the HQ Clinic in Toronto, Canada. HQ Clinic provides services to at-risk populations in the Toronto area, with 2024 self-reported CT prevalence of 2.11% and NG prevalence of 2.22%. Following clinical testing (using the Aptima test), de-identified leftover urine from 160 specimens (60 negative, 50 positive for CT, and 55 positive for GC [including 5 dual CT/NG-positive]) was aliquoted into 15 mL tubes, frozen at −20°C, and shipped to NML for further processing. At arrival, samples were stored at −80°C. Ten specimens were thawed per day and processed as per the outline shown in Fig. S5. In addition to the standard DUS preparation method (referred to as DUS-A), we evaluated two additional conditions: (i) placing the DUS directly into a storage bag with two desiccants (bypassing the overnight drying requirement, referred to as DUS-B); and (ii) storing the DUS for 3 weeks at RT prior to elution and testing (referred to as DUS-C).

A separate pilot study was performed using DUS strips received from the British Columbia Centre for Disease Control laboratory (BCCDC, Vancouver). For the BCCDC clinic, the self-reported prevalence in 2024 for CT is 4.0% for males and 3.4% for females and for NG is 1.0% for males and 0.3% for females. Residual urine samples from clinical testing (using the Aptima Combo 2 Assay) were applied to the DUS strip at the BCCDC laboratory, dried overnight, stored at RT, and shipped weekly to the NML for testing. Upon arrival, samples were eluted, filter paper was removed, and samples were stored at −20°C until batch testing. This portion of the study included 60 negative samples, 33 CT-positive samples, and 11 NG-positive samples.

No clinical or demographic data were available for study participants. All testing was performed blinded to the initial clinic test results.

### CT genotyping

Genotyping was performed via nested PCR and Sanger sequencing of the *ompA* gene as previously described (19). Sequences were aligned using the Basic Local Alignment Search Tool (BLAST) from the National Center for Biotechnology Information (NCBI) website (20). Identity scores were compared between DUS and urine.

### Molecular typing by NG-MAST

Gonorrhea-positive samples were sequence typed using a well-established, previously described, *Neisseria gonorrhoeae* Multi-Antigen Sequence Typing (NG-MAST) PCR-based method (21, 22). Briefly, extracted DNA underwent nested PCR reactions, targeting the *porB* and *tbpB* genes. Second round products were then sequenced by Sanger sequencing and the *porB* and *tbpB* allele types were assigned using the PubMLST database (23). NG-MAST sequence types (STs) were identified.

### Statistical analysis

Statistical analysis was performed using GraphPad Prism version 10.4.1 (La Jolla, CA, USA). In order to eliminate the effects of freeze-thawing on test performance characteristics, the Aptima test was run on all HQ urine samples post-thawing at the NML; samples with discordant results were removed from the analysis. For BCCDC samples, test performance was calculated with reference to the original Aptima test results obtained at BCCDC. Wilson’s score method was used to calculate 95% confidence intervals (CIs) to assess levels of uncertainty induced by sample size. To measure agreement between assays, Cohen’s kappa coefficient was calculated (24). Due to values in the equivocal range of the Aptima test, two sets of analyses were performed: one considering DUS equivocal values as positive, and the second considering DUS equivocal values as negative. A simple *t*-test or ANOVA with Tukey’s post-test was used to calculate differences in RLU (1,000×) and Cq values between groups (*P* value < 0.05 was considered statistically significant). For RT-PCR negative values, a Cq value of 40 was assigned. NG-MAST was only performed on NG positive samples by NML Aptima testing. Pearson correlation was performed to assess the correlation between different sample types for Cq and RLU (1,000×) values.

## RESULTS

### DUS performance using bacterial cultures

**Fig 2:**
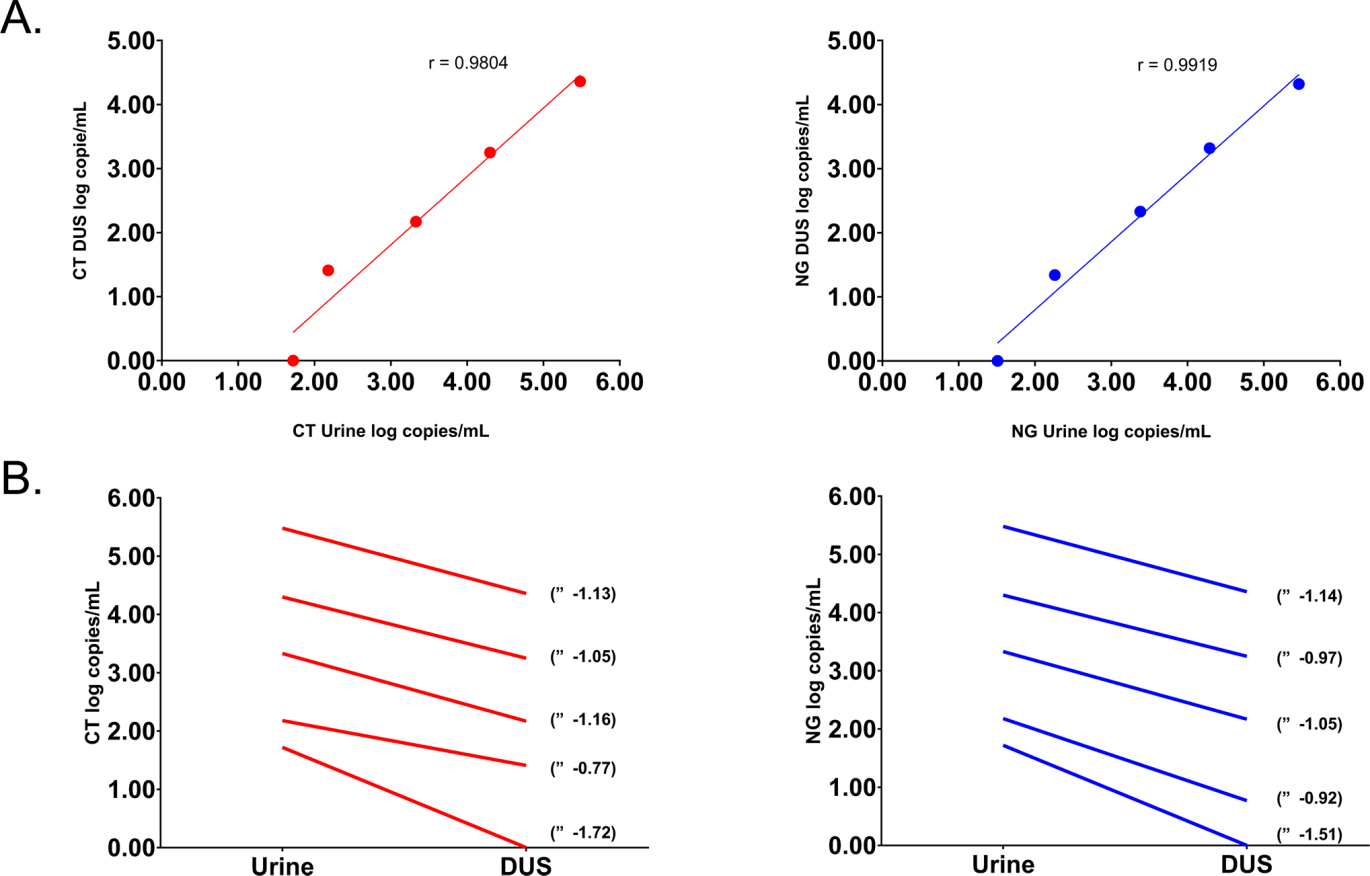
CT (red) and NG (blue) copies/mL measurements of bacterial stock dilutions applied to urine and DUS. (**A**) Pearson correlation between log copies/mL for urine and DUS for bacterial stock concentrations with measurable results for both sample types (10^3^–10^7^). Linear regression line added for visualization purposes. (**B**) Difference in log copies/mL between urine and DUS for bacterial stock concentrations with measurable results for both sample types (10^3^–10^7^). Supporting data for this figure can be found in Table S1. Experiments were performed using the Whatman 903 filter paper strip.

Following elution of the DUS with 1.5 mL of STM, approximately 900 µL of liquid was available for testing, with the rest retained by the filter paper. In order to assess specimen loss due to filter paper sample retention, we applied prepared bacterial dilutions to strips and urine and calculated the resulting copies/mL using our in-house qPCR methods (Fig. 2; Table S1). We observed a strong correlation between the obtained values from DUS and urine for both CT and NG (CT: *r* = 0.9804, NG: *r* = 0.9919, Fig. 2A). At bacterial stock concentrations with measurable results for both DUS and urine (10^3^–10^7^), DUS resulted in an average of 1.17 log loss in CT copies/mL and 1.12 log loss in NG copies/mL compared to urine (Fig. 2B).

**Fig 3:**
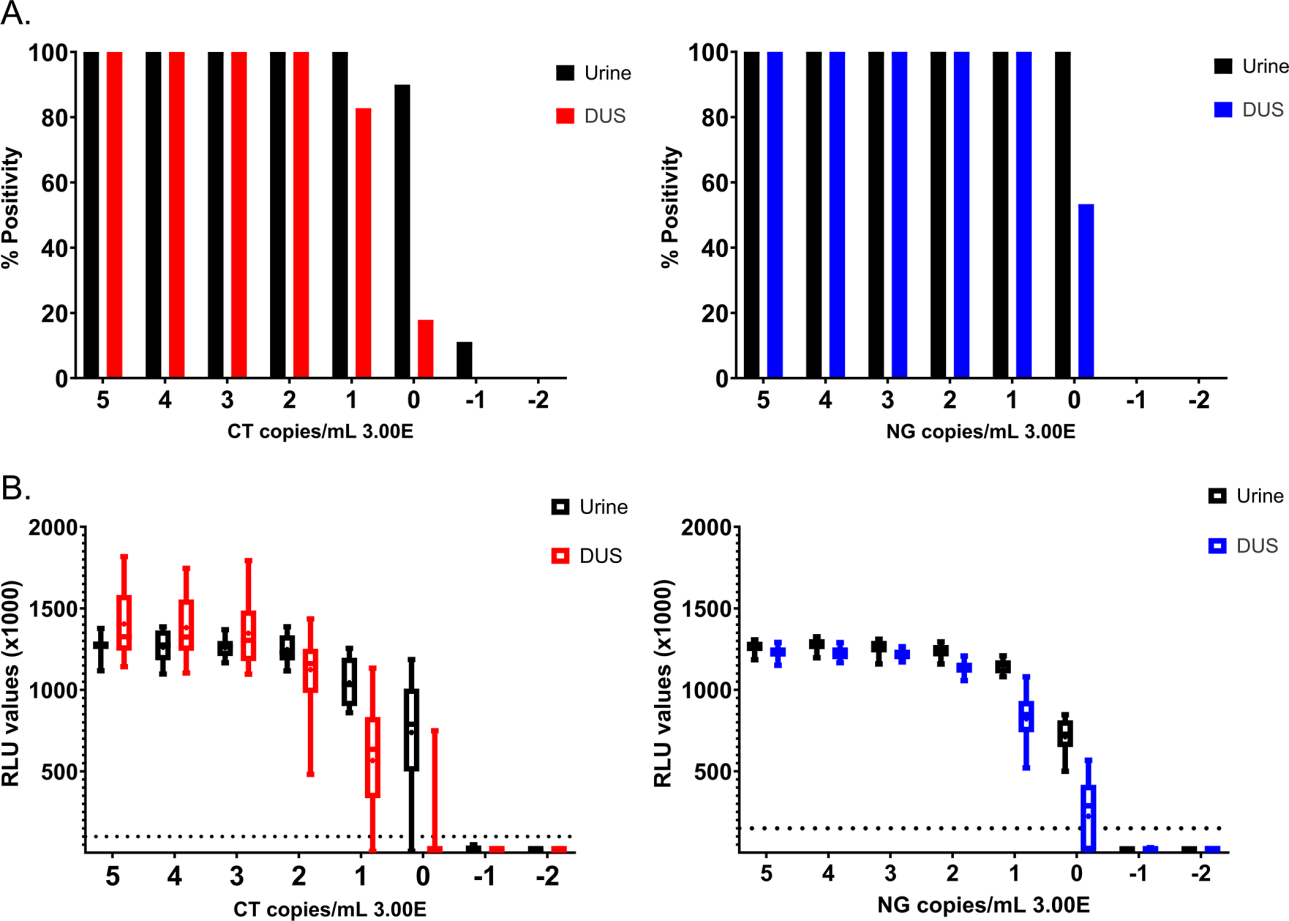
Aptima Assay performance for urine and DUS spiked with CT and NG bacterial culture. (**A**) The percent detectability for urine and DUS across tested bacterial dilutions. (**B**) RLU (1,000×) values across tested bacterial dilutions. Box plot with max and min whiskers. Dashed line indicates positive RLU (1,000×) threshold (CT = 100; NG = 150). One false positive measurement (643 RLU [1,000×]) for CT at 3.00E−02 was removed from the graph (contamination occurred due to lab error during samples preparation, highlighted in Table S2). Experiments were performed using the Whatman 903 filter paper strip.

Next, we evaluated the performance of DUS on the Aptima test using the prepared bacterial stocks. We observed 100% positivity for urine and DUS samples with CT dilutions from 3 × 10^5^ to 3 × 10^2^ copies/mL (Fig. 3A; Table S2). At 30 CT copies/mL, DUS positivity dropped to 83%, while staying at 100% for urine. At 3 copies/mL, the percent positivity for urine dropped to 90% and to 18% for DUS. For NG, both urine and DUS maintained 100% positivity for sample dilutions between 3 × 10^5^ and 30 copies/mL. At 3 NG copies/mL, we observed 100% positivity for urine while DUS dropped to 53% (Fig. 3A; Table S3). Similar observations can be seen with regards to RLU (1,000×) values (Fig. 3B; Table S2 and S3) with DUS values falling below detectability at 30 copies/mL for CT and 3 copies/mL for NG.

### DUS stability

**Fig 4:**
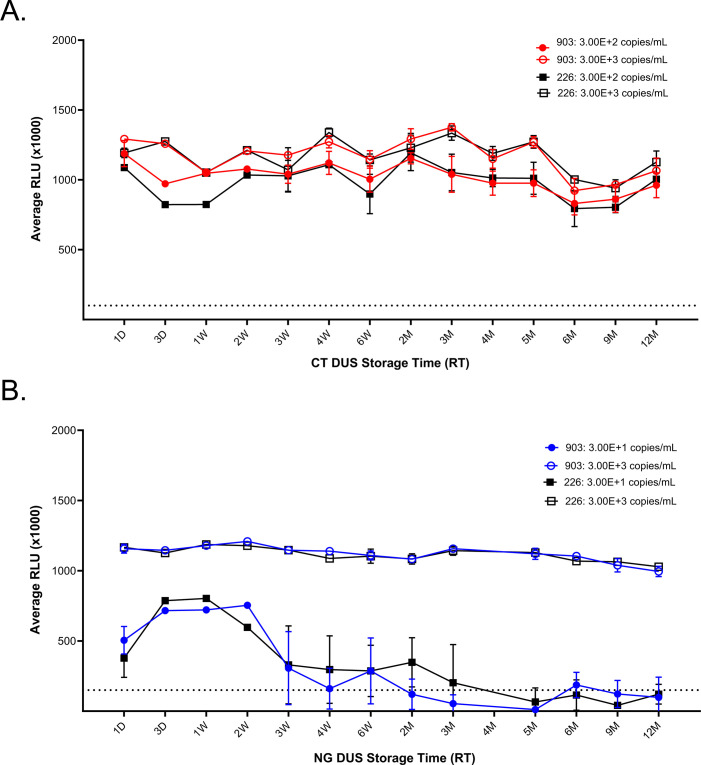
DUS stability at room temperature (RT) for (**A**) CT and (**B**) NG over the 12-month period. Aptima Assay RLU (1,000×) values (mean and standard deviation [SD]) are indicated for samples collected on Whatman 903 (circle) and A-226 (square) filter papers at 3.00E+3 copies/mL for both CT and NG (unfilled circles and squares), and 3.00E+2 for CT and 3.00E+1 for NG (filled circles and squares). D, day; M, month; W, week. Dashed line indicates positive RLU (1,000×) threshold (CT = 100; NG = 150).

We tested the stability of the DUS samples for short-term and long-term storage at different temperatures. Strips were stored at indicated time intervals for up to 12 months and analyzed using the Aptima test (Fig. 4; Fig. S6). We tested two bacterial concentrations, a higher concentration where we did not observe any difference in RLU (1,000×) values between urine and DUS (3 × 10^3^ copies/mL for both CT and NG) and a lower concentration where we first observed a drop in RLU (1,000×) values for DUS while still maintaining 100% detectability (3 × 10^2^ copies/mL for CT and 30 copies/mL for NG, Fig. 3). At higher concentrations, we observed no loss in CT and NG detectability, with RLU (1,000×) values above the detection limit at all storage conditions (RT, +30°C, +4°C, −20°C, and −80°C) for up to 12 months (Fig. 4; Fig. S6). A similar trend was observed for CT at 3 × 10^2^ copies/mL. At 30 copies/mL for NG, we saw a drop in RLU (1,000×) values at 3 weeks for RT, +4°C, −20°C, and −80°C, and at first measure (day 3) for +30°C, indicating DUS sample instability at lower bacterial loads when stored for more than 2 weeks. No difference in RLU (1,000×) values was observed between two filter paper types.

### DUS performance using clinical specimens

We examined the performance of DUS for CT and NG testing using clinical specimens. One NG-positive and two CT-positive samples from HQ tested negative on the Aptima test following sample freezing and thawing (Table S4) and were excluded from analysis. The Aptima test results for CT using urine and DUS specimens were in high agreement with Cohen’s *κ* > 0.95 (Table S5). This remained true across all DUS preparation conditions and analysis types (equivocal samples considered as positive and negative). In comparison to urine, all CT DUS types had >95% sensitivity and 100% specificity when equivocal samples were considered positive (Table 1). Similar results were obtained when equivocal samples were considered negative (Table S5). When looking at the CT RLU (1,000×) values obtained for different sample types, we observed significantly higher RLU (1,000×) values for DUS-C samples compared to urine (*P* < 0.05) and DUS-B (*P* < 0.05, Fig. S7A). Finally, there was a strong correlation in RLU (1,000×) values between all sample types (*r* > 0.70, *P* < 0.0001, Fig. S7B).

**TABLE 1:** Diagnostic accuracy (sensitivity and specificity, 95% CI) of the A-226 DUS in reference to urine in clinical samples obtained from the HQ Clinic in Toronto, tested by Aptima Assay^*a*^^,^^*b*^^,^^*c*^^,^^*d*^^,^^*e*^

	Pathogen	True positive	False positive	False negative	True negative	Sensitivity (95% CI)	Specificity (95% CI)
DUS-A	CT	48	0	0	109	100.00 (92.59–100.00)	100.00 (96.60–100.00)
	NG	52	1	2	102	96.30 (87.46–98.98)	99.03 (94.70–99.83)
DUS-B	CT	47	0	1	109	97.92 (89.10–99.63)	100.00 (96.60–100.00)
	NG	52	0	2	103	96.30 (87.46–98.98)	100.00 (96.40–100.00)
DUS-C	CT	46	0	2	109	95.83 (86.02–98.85)	100.00 (96.60–100.00)
	NG	53	0	1	103	98.15 (90.23–99.67)	100.00 (96.40–100.00)

^*a*^ DUS-A (A-226 DUS dried overnight prior to storage).

^*b*^ DUS-B (A-226 DUS placed directly into the sample collection bag with two desiccants).

^*c*^ DUS-C (A-226 DUS dried overnight and stored at RT for 3 weeks prior to elution).

^*d*^ Equivocal values were treated as positive.

^*e*^ Extended analysis can be found in Tables S5 and S6.

Similar results were observed for NG, with high agreement between urine and different DUS sample types (Cohen’s *κ* > 0.94, Table S6). In reference to urine, all DUS sample types had >96% sensitivity and >99% specificity (equivocal = positive, Table 1). No significant difference was observed in NG RLU (1,000×) values between different sample types (Fig. S7A). There was a strong correlation between RLU (1,000×) values between all sample types (*r* > 0.70, *P* < 0.0001, Fig. S7B).

Similar performance was observed in the BCCDC pilot study (Table S7). For CT, DUS sensitivity compared to urine samples was 96.97% (95% CI: 84.68–99.46) with specificity of 100.00% (95% CI; 94.87–100.00). For NG, DUS sensitivity compared to urine samples was 100.00% (95% CI: 75.75–100.00) with specificity of 98.91% (95% CI: 94.10–99.81). No significant difference was observed in RLU (1,000×) values between urine and DUS for either CT or NG (Fig. S8).

### Suitability of DUS for downstream pathogen characterization

To evaluate the suitability of DUS specimen for further pathogen characterization, all CT-positive and NG-positive samples were processed using established NML protocols.

**Fig 5:**
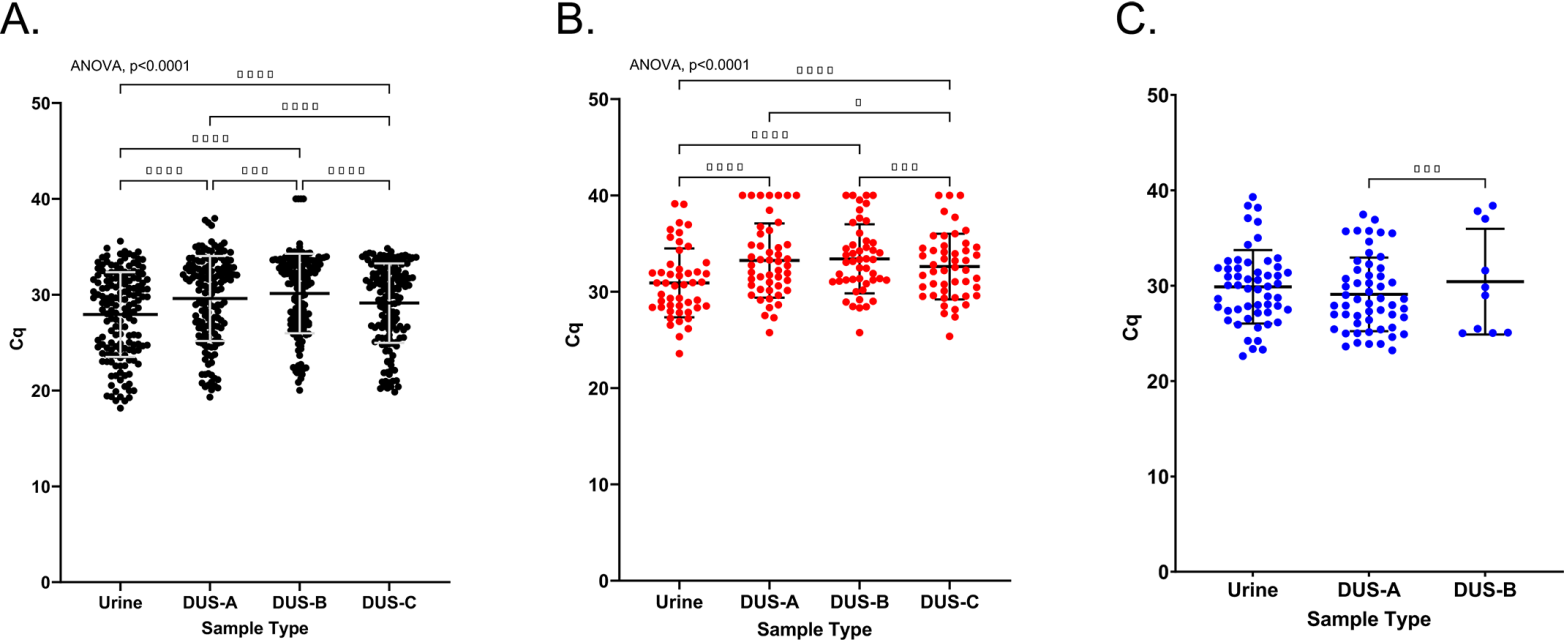
Cq values for (**A**) RNAseP target (all samples, *N* = 157), (**B**) CT Cryptic plasmid target (CT-positive samples, *N*=48), and (**C**) NG *porA* target (NG-positive samples, *N* = 54). Cq of 40 was input (max cycle number) for all RT-PCR negative results. Figure bars indicate mean and SD. *P < 0.05, **P < 0.01, ***P < 0.001, and ****P < 0.0001. DUS-A (A-226 DUS dried overnight prior to storage), DUS-B (A-226 DUS placed directly into the sample collection bag with two desiccants), and DUS-C (A-226 DUS dried overnight and stored at RT for 3 weeks prior to elution).

All urine and DUS samples (A, B, and C, *n* = 157 per sample type) were analyzed using an in-house CT RT-PCR assay, routinely used for detection of CT and LGV differentiation. With regard to CT detection, we observed a high degree of concordance between all DUS sample types and urine, with Cohen’s *κ* > 0.92 and >91% sensitivity and >98% specificity (Table S8). In order to assess loss in genomic material due to DUS sample processing, we compared Cq values of RNaseP (host gene target) for all specimens (*n* = 157) between DUS and urine, as well as the CT cryptic plasmid target for samples that tested CT-positive by the Aptima test (*n* = 48). Overall, we observed statistically significant differences in Cq values between different sample types for both RNaseP (ANOVA *P* < 0.0001) and CT cryptic plasmid targets (ANOVA *P* < 0.0001, Fig. 5A and B). For the RNaseP target, all DUS sample types had significantly higher Cq values compared to urine, indicating some loss of genomic material. Between DUS sample types, DUS-B had significantly higher RNAseP Cq values compared to DUS samples dried overnight prior to storage (DUS-A: *P* < 0.01, and DUS-C: *P* < 0.001). Interestingly, we observed significantly lower RNAseP Cq values in DUS-C compared to DUS-A (*P* < 0.0001). Similar outcomes were observed with CT cryptic plasmid target (Fig. 5B), with Cq values significantly higher for all DUS sample types compared to urine (*P* < 0.0001). DUS-C had significantly lower Cq values compared to the other strips (DUS-A: *P* < 0.05, DUS-B: *P* < 0.001). There was a strong significant correlation in Cq values for both RNAseP and CT cryptic plasmid between all sample types (*r* > 0.90, *P* < 0.0001 across all comparisons, Fig. S9A and B).

Due to sample limitation, NG RT-PCR for the *porA* target was performed on all NG-positive urine and paired DUS-A samples (*n* = 54) and a subset of DUS-B samples (*n* = 10). We observed high sensitivity for both DUS-A and DUS-B compared to urine (100.00%, Table S9). We observed no significant difference in Cq values between urine and DUS samples for the NG *porA* target (Fig. 5C). The DUS-B sample subset had significantly higher Cq values compared to DUS-A (*P* < 0.001). We observed a strong significant correlation in Cq values for NG *porA* between different sample types (*r* > 0.75, *P* < 0.0001, Fig. S9C)

With regards to the CT *ompA* sequencing results (Table S10 and S11), we observed some loss in percent sensitivity with the DUS sample types compared to urine (DUS-A; 78.05% [95% CI: 63.29–88.00], DUS-B: 69.05% [95% CI: 53.97–80.93]; DUS-C: 71.43% [95% CI: 56.43–82.83]). Between samples that generated sequence results, there was 100% concordance between identified CT genotypes.

For NG-MAST, 50 out of 54 samples (92.59%) were successfully typed identifying 29 unique STs. This resulted in high percent sensitivity for DUS- A (96.00% [95% CI: 86.54–98.90]) and DUS-B (80.00% [95% CI: 49.02–94.33]) in reference to urine (Tables S12 and 13). There was 100% agreement in STs between urine, DUS-A, and DUS-B.

## DISCUSSION

Here, we showed that the developed filter paper strips represent an accurate, stable, and convenient urine sample collection method for CT and NG diagnostic testing. The filter paper strips were designed in a manner that would simplify sample collection, minimize processing, and be compatible with diagnostic methods commonly used in laboratories for high throughput CT and NG testing.

With clinical samples, we observed a high degree of diagnostic accuracy (>91% sensitivity and specificity), compared to urine across all evaluated DUS preparation and storage methods. As indicated by the Cq values for host genomic and CT bacterial targets, we did observe a loss in genomic material with DUS compared to urine, likely due to sample retention by the filter paper strip following elution. This is in concordance with our experiments using bacterial stocks, where we see an approximate 1 log drop in copies/mL from DUS compared to urine specimens. However, the observed loss in genomic material in DUS elute did not influence Aptima or RT-PCR test performance, as indicated by the high diagnostic accuracy between urine and all DUS preparation methods.

When testing bacterial culture dilutions using the Aptima test, DUS started to lose sensitivity at 30 copies/mL for CT and 3 copies/mL for NG. A previous community-based study reported a geometric mean chlamydial load in first void urine of 446 copies/mL (95% CI: 219–916 copies/mL) in women and 626 copies/mL (95% CI: 156–2,515 copies/mL) in men (25), well above the concentration where a loss in DUS sensitivity was observed. Similarly, the mean NG loads in male and female clinical urine specimens are reported to be 4.5 ± 1.0 log_10_ CFU/mL (26), well above the 30 copies/mL where we start observing a loss in DUS sensitivity.

Concerning sample stability, DUS samples at higher concentrations showed long-term stability with no drop in RLU (1,000×) values when analyzed using the Aptima test at all tested temperatures (−80°C, −20°C, +4°C, RT, and +30°C) for up to 12 months. At lower concentrations (3 copies/mL for NG), we observed sample instability, with RLU (1,000×) values dropping below the limit of detection following 2 weeks of storage at tested temperatures. The results show that DUS are stable for at least 2 weeks at room temperature, irrespective of bacterial load, which provides enough time for mail-in shipment or delayed shipping to the lab for diagnostic testing. Additionally, DUS samples are stable for prolonged periods at various temperature storage conditions, making them suitable for retrospective epidemiological studies. In addition to routine screening and testing, the developed and evaluated method of urine sample collection would benefit research studies, especially with regard to storage requirements (space and need for freezers) and can lead to a major reduction in costs of sample shipping, in comparison to liquid urine collection.

While CT bacterial load results indicated that drying the strip overnight was superior to drying the strip in the sample collection bag with an additional desiccant, the overnight drying step would likely be a major barrier for self-sample collection, specifically concerning privacy and convenience. Despite some loss in bacterial load, placing the strip directly into the sample collection bag did not negatively impact the diagnostic performance on the Aptima test (targeting rRNA) nor the in-house methods (targeting DNA) for CT or NG detection. To further improve the stability and recovery of genomic material, use of higher capacity desiccants can be considered when eliminating the overnight drying step.

Interestingly, the DUS-C samples that were stored in storage bags for 3 weeks showed increased recovery of CT and host genomic material compared to other DUS sample types. This was observed with both the Aptima test and the in-house RT-PCR assay with higher RLU (1,000×) and lower Cq values (RNAseP and cryptic plasmid targets). It is likely that prolonged storage allowed for additional removal of residual humidity and therefore increased sample recovery during the elution process.

In addition to suitability for CT and NG detection, our results indicate that the DUS sample is suitable for downstream pathogen characterization. While we did observe some loss of sensitivity with the DUS compared to urine with regards to CT genotyping, this is expected in samples with high Cq values and is commonly observed during routine diagnostic testing. In samples where we obtained a sequence, we observed 100% concordance between urine and DUS sequencing results for CT and NG, demonstrating that DUS eluates can be used for organism typing and other genetic analysis.

Rather than serving as a replacement for established self-collection methods—such as first-void urine for men or vaginal swabs for women—DUS provide a complementary option that may be valuable in settings where the transport or handling of liquid urine is impractical. A dry, nonliquid sample reduces biosafety and packaging requirements, eliminates leakage concerns, and facilitates mail-in or community-based testing models, particularly in remote regions where certified couriers for biological liquids are limited or costly. In addition, DUS can be integrated with dried blood spot cards within a single low-burden collection kit, supporting broader STI testing through streamlined transport and simplified logistics.

One of the main limitations of the presented study is that strip preparation and testing were done retrospectively, in a controlled laboratory environment. Further studies are needed to evaluate the suitability and barriers of utilizing DUS in real-life community settings. Practical challenges such as contamination risk and variable user handling have not yet been evaluated and should be addressed in future studies. While the full demographics of the study population were not available, due to the nature of the study clinics, the data presented here pertains to urine samples primarily obtained from male participants. Additional evaluation of the sample collection methodology is recommended for the female population. Overall, while the DUS offers several potential advantages—such as simplified transportation, compatibility with decentralized testing models, and integration with dried blood specimens—its successful implementation will require careful evaluation of real-world handling and usability.

The global increase in STI rates poses a significant public health challenge and necessitates the need for improved testing and screening strategies. Our study demonstrates that the developed DUS represents an accurate and convenient method for CT and NG detection and characterization with potential to increase STI screening coverage and reduce global STI rates.

## Data Availability

Additional data sets (not provided in the supplemental materials) generated during the current study are available from the corresponding author on reasonable request.

## SUPPLEMENTAL MATERIAL

Supplemental tables and figures jcm.01618-25-s0001.pdf Tables S1 to S12 and Figures S1 to S9.

ASM does not own the copyrights to Supplemental Material that may be linked to, or accessed through, an article. The authors have granted ASM a non-exclusive, world-wide license to publish the Supplemental Material files. Please contact the corresponding author directly for reuse.
