# Supplementary material for: Development and evaluation of dried urine strip for genital chlamydia and gonorrhea testing
Source: J Clin Microbiol. 2026 Apr 2;64(5):e01618-25. doi: 10.1128/jcm.01618-25 (PMC13170174; doi:10.1128/jcm.01618-25)
Supplement: Supplemental tables and figures — Tables S1 to S12 and Figures S1 to S9. [file jcm.01618-25-s0001.pdf]

Supplementary Appendix

Development and evaluation of dried urine strip for genital chlamydia and gonorrhea testing

Suzanne Gibbons[1], Clarissa Klenke[1,2], Felicia Roy[1], Vanessa Schulz[1], Kimberly Ta[1], Sharon Simon[1], Jennifer Beirnes[1], Sabaparvin Shaikh[1], Ravinder Lidder[1], Christine Mesa[1], Shelley Peterson[1], Virginia Resh[3], Janet Fung[3], Jennifer Grant[3], Olivia Gemmell[4], Kevin Woodward[4,5], Paul Sandstrom[1,2], Alberto Severini[2], Irene Martin[1], John Kim[1], Aida Sivro[1,2,5]

[1]National Microbiology Laboratory Branch, Public Health Agency of Canada, Winnipeg, Manitoba, Canada

[2]Department of Medical Microbiology and Infectious Diseases, Faculty of Health Sciences, University of Manitoba, Winnipeg, Manitoba Canada

[3]BCCDC Public Health Laboratory, BC Centre for Disease Control, Vancouver, British Columbia, Canada

[4]HQ Toronto, Toronto, Ontario, Canada

[5]Department of Medicine, Division of Infectious Diseases, McMaster University, Ontario, Canada

[6]Department of Medical Microbiology, University of KwaZulu-Natal, Durban, South Africa

Corresponding author: Aida Sivro (sivroaida@gmail.com);

Supplemental Appendix Table of Contents:

Supplemental Table 1. Data and calculations for copies/mL detection of CT and NG stock dilutions yielding measurable results in urine and DUS measured by qPCR.

Supplemental Table 2. Data and calculations for Aptima Combo 2® measurements of CT bacterial stock dilutions.

Supplemental Table 3. Data and calculations for Aptima Combo 2® measurements of NG bacterial stock dilutions.

Supplemental Table 4. Aptima Combo 2® Results for 3 specimens excluded from the analysis.

Supplemental Table 5. Diagnostic accuracy of the A-226 DUS in reference to urine for detection of CT in clinical samples obtained from HQ Clinic in Toronto.

Supplemental Table 6. Diagnostic accuracy of the A-226 DUS in reference to urine for detection of NG in clinical samples obtained from HQ Clinic in Toronto.

Supplemental Table 7. Diagnostic accuracy of the A-226 DUS in reference to urine for detection of CT and NG in clinical samples obtained from BCCDC.

Supplemental Table 8. Diagnostic accuracy of the A-226 DUS in reference to urine for detection of CT in clinical samples obtained from HQ Clinic in Toronto, tested by CT RT-PCR.

Supplemental Table 9. Diagnostic accuracy of the A-226 DUS in reference to urine for detection of NG in clinical samples obtained from HQ Clinic in Toronto, tested by porA RT-PCR.

Supplemental Table 10. Chlamydia genotyping results indicating samples that were sequenced and genotype agreement between urine and DUS samples.

Supplemental Table 11. 2x2 table for sensitivity and specificity calculations for CT sequencing in urine and DUS samples.

Supplemental Table 12. Gonorrhea sequence typing results indicating samples that were sequenced and sequence type agreement between urine and DUS samples.

Supplemental Table 13. 2x2 table for sensitivity calculations for NG sequencing in urine and DUS samples.

Supplemental Figure 1. DUS storage bag.

Supplemental Figure 2. DUS preparation.

Supplemental Figure 3. DUS elution.

Supplemental Figure 4. Diagram of the DUS evaluation using the prepared CT and NG bacterial stock dilutions.

Supplemental Figure 5. Study diagram for the DUS clinical validation using specimens obtained from the HQ Clinic in Toronto.

Supplemental Figure 6. DUS stability at +4ºC, -20ºC, +30ºC and -80ºC for A. CT and B. NG over 12 month period.

Supplemental Figure 7. RLU (x1000) values for CT and NG for different sample collection methods and conditions and heatmap of correlations between RLU values obtained for different sample types.

Supplemental Figure 8. BCCDC pilot study RLU (x1000) values for urine and DUS for CT and NG.

Supplemental Figure 9. Heatmap of RT-PCR Cq correlations for RNAseP , CT Cryptic plasmid and RT-PCR NG correlations for *porA* targets between different sample types.

Supplemental Table 1. Data and calculations for copies/mL detection of CT and NG stock dilutions yielding measurable results in urine and DUS by qPCR.

Stock dilutions copies/mL	Expected copies / qPCR test (2µL of stock dilution*)	Sample type	Actual copies per qPCR test				Average actual copies /qPCR test (2µL of stock dilution)	Copies/400µL spiked urine (10µL of stock dilution)	Copies/mL spiked urine		Urine - DUS
			Replicate 1	Replicate 2	Replicate 3	Replicate 4			Extrapolated	Log_{10}	delta Log_{10}
**Stock CT dilutions**		**Urine spiked with stock CT dilutions**									
1.00E+07	2.00E+04	urine	2.99E+04	3.01E+04	1.84E+04	1.85E+04	2.42E+04	1.21E+05	3.03E+05	5.48	1.13
		DUS	1.97E+03	1.92E+03	1.67E+03	1.69E+03	1.81E+03	9.06E+03	2.27E+04	4.36	
1.00E+06	2.00E+03	urine	1.75E+03	1.87E+03	1.34E+03	1.48E+03	1.61E+03	8.05E+03	2.01E+04	4.30	1.05
		DUS	1.30E+02	1.66E+02	1.70E+02	1.05E+02	1.43E+02	7.14E+02	1.78E+03	3.25	
1.00E+05	2.00E+02	urine	1.90E+02	1.99E+02	1.48E+02	1.55E+02	1.73E+02	8.65E+02	2.16E+03	3.33	1.16
		DUS	1.42E+01	1.12E+01	1.32E+01	9.08E+00	1.19E+01	5.96E+01	1.49E+02	2.17	
1.00E+04	2.00E+01	urine	8.62E+00	1.08E+01	1.61E+01	1.26E+01	1.20E+01	6.02E+01	1.50E+02	2.18	0.77
		DUS	neg.	neg.	2.05E+00	neg.	2.05E+00	1.03E+01	2.56E+01	1.41	
1.00E+03	2.00E+00	urine	4.35E+00	3.81E+00	4.42E+00	neg.	4.19E+00	2.10E+01	5.24E+01	1.72	1.72
		DUS	neg.	neg.	neg.	neg.	NA	NA	NA	NA	
**Stock NG Dilutions**		**Urine spiked with stock NG dilutions**									
1.00E+07	2.00E+04	urine	1.57E+04	1.38E+04	3.06E+04	3.12E+04	2.28E+04	1.14E+05	2.85E+05	5.46	1.13
		DUS	2.25E+03	1.97E+03	1.15E+03	1.39E+03	1.69E+03	8.45E+03	2.11E+04	4.32	
1.00E+06	2.00E+03	urine	1.43E+03	1.52E+03	1.58E+03	1.67E+03	1.55E+03	7.75E+03	1.94E+04	4.29	0.97
		DUS	2.16E+02	2.01E+02	1.28E+02	1.18E+02	1.66E+02	8.29E+02	2.07E+03	3.32	
1.00E+05	2.00E+02	urine	1.65E+02	1.44E+02	1.91E+02	2.60E+02	1.90E+02	9.50E+02	2.38E+03	3.38	1.05
		DUS	2.38E+01	2.08E+01	1.32E+01	9.88E+00	1.69E+01	8.46E+01	2.12E+02	2.33	
1.00E+04	2.00E+01	urine	1.76E+01	1.94E+01	9.04E+00	1.18E+01	1.45E+01	7.23E+01	1.81E+02	2.26	0.92
		DUS	1.75E+00	neg.	1.75E+00	1.75E+00	1.75E+00	8.75E+00	2.19E+01	1.34	
1.00E+03	2.00E+00	urine	2.28E+00	1.75E+00	1.75E+00	4.60E+00	2.60E+00	1.30E+01	3.24E+01	1.51	1.51
		DUS	Cq > 40	neg.	Cq > 40	neg.	NA	NA	NA	NA	

Cq = quantitation cycle; NA = not applicable; Neg= negative

*400 µL of urine/DUS elute used for DNA extraction is expected to contain 10 µL of the original bacterial stock dilution. DNA was eluted in 50 µL and 10 µL (1/5) of the DNA was used for qPCR. Assuming 100% recovery of the input DNA this would result in 2 µL (10 µL /5) of original bacterial stock per qPCR test.

Supplemental Table 2. Data and calculations for Aptima Combo 2® measurements of CT bacterial stock dilutions.

Copies/ mL	Copies/ mL	sample type	Batch #1						Batch #2						Batch #3						Batch #4						Batch #5						RLU (X1000)		Positivity Rate
			1	2	3	4	5	6	1	2	3	4	5	6	1	2	3	4	5	6	1	2	3	4	5	6	1	2	3	4	5	6	Avg	SD	
Stock CT dilutions	Prepared sample#	Chlamydia Panther Measurements [RLU (x1000)]																																	
1.00E+07	3.00E+05	urine	1260	1377	NA	NA	NA	NA	1272	1285	NA	NA	NA	NA	1117	1281	NA	NA	NA	NA	1273	1223	NA	NA	NA	NA	1310	1293	NA	NA	NA	NA	1269	66	10/10 (100%)
		DUS	1748	1740	1568	1656	1459	1407	1807	1817	1626	1666	1523	1464	1217	1183	1244	1216	1320	1261	1298	1273	1142	1291	1214	1163	1398	1332	1242	1366	1267	1207	1404	208	30/30 (100%)
1.00E+06	3.00E+04	urine	1310	1377	NA	NA	NA	NA	1097	1202	NA	NA	NA	NA	1197	1136	NA	NA	NA	NA	1275	1275	NA	NA	NA	NA	1386	1361	NA	NA	NA	NA	1262	101	10/10 (100%)
		DUS	1611	*	1567	1586	1518	1494	1704	1746	1632	1665	1487	1488	1270	1262	1143	1142	1103	*	1168	1339	1268	1237	1199	1249	1221	1326	1341	1307	1323	1292	1382	189	28/28 (100%)
1.00E+05	3.00E+03	urine	1369	1325	NA	NA	NA	NA	1214	1189	NA	NA	NA	NA	1270	1277	NA	NA	NA	NA	1214	1166	NA	NA	NA	NA	1292	1261	NA	NA	NA	NA	1258	63	10/10 (100%)
		DUS	1728	1697	1572	*	1472	1398	1793	1760	1591	1501	1363	1308	1124	1100	1196	1167	1151	1120	1304	1186	1278	1252	1096	1285	1323	1308	1302	1316	1131	1262	1348	208	29/29 (100%)
1.00E+04	3.00E+02	urine	1367	1387	NA	NA	NA	NA	1194	1157	NA	NA	NA	NA	1116	1189	NA	NA	NA	NA	1278	1209	NA	NA	NA	NA	1324	1249	NA	NA	NA	NA	1247	91	10/10 (100%)
		DUS	1281	1064	1372	*	1223	906	1435	1428	1345	1297	1297	1120	906	951	972	1174	1076	988	1072	1161	1163	1161	1128	917	1195	1199	1133	1174	975	481	1124	195	29/29 (100%)
1.00E+03	3.00E+01	urine	1255	966	NA	NA	NA	NA	1006	872	NA	NA	NA	NA	1092	910	NA	NA	NA	NA	1059	1188	NA	NA	NA	NA	860	1223	NA	NA	NA	NA	1043	145	10/10 (100%)
		DUS	916	639	10	13	370	11	150	635	9	644	724	635	809	606	760	768	570	453	919	551	581	*	1020	1133	857	976	302	871	484	21	567	335	24/29 (83%)
1.00E+02	3.00E+00	urine	732	1186	NA	NA	NA	NA	795	784	NA	NA	NA	NA	373	10	NA	NA	NA	NA	953	843	NA	NA	NA	NA	1172	542	NA	NA	NA	NA	739	358	9/10 (90%)
		DUS	7	9	8	9	8	*	7	9	8	10	7	14	10	9	13	542	12	10	13	12	559	566	460	749	11	10	14	11	11	*	111	224	5/28 (18%)
1.00E+01	3.00E-01	urine	14	12	NA	NA	NA	NA	13	14	NA	NA	NA	NA	13	984	NA	NA	NA	NA	50	11	NA	NA	NA	NA	15	13	NA	NA	NA	NA	114	306	1/9 (11%)
		DUS	7	13	10	*	9	11	6	6	10	9	10	10	11	11	10	13	14	10	10	10	12	11	13	12	10	14	11	11	14	13	11	2	0/29 (0%)
1.00E+00	3.00E-02	urine	8	10	NA	NA	NA	NA	9	10	NA	NA	NA	NA	9	9	NA	NA	NA	NA	8	16	NA	NA	NA	NA	9	13	NA	NA	NA	NA	10	3	0/10 (0%)
		DUS	8	8	643	*	10	*	9	6	7	14	11	10	8	15	10	10	10	11	11	*	8	17	16	13	11	10	9	26	18	13	35	122	1/27 (4%)

RLU = Relative light unit; SD = standard deviation; Avg = average; NA = not applicable

Panther CT positive = >100 Total RLU (x1000); Panther Equivocal region CT only = 25 to <100 Total RLU (x1000); Panther Equivocal region CT/GC dual = 85 to <250 Total RLU (x1000)

* = instrument error RDFS/VVFS; invalid result (RDFS; Sample dispense verification failure/ VVFS; Volume verification failure)

Red font = Known contamination due to lab error during sample preparation. Sample was excluded from the analysis.

#Concentration resulting from addition of 120µl of bacterial stock dilution to 4ml of urine used to prepare DUS and urine samples for testing.

Supplemental Table 3. Data and calculations for Aptima Combo 2® measurements of NG bacterial stock dilutions.

copies/ mL	copies/ml	sample type	Batch #1						Batch #2						Batch #3						Batch #4						Batch #5						RLU (x1000)		Positivity Rate
			1	2	3	4	5	6	1	2	3	4	5	6	1	2	3	4	5	6	1	2	3	4	5	6	1	2	3	4	5	6	Avg	SD	
Stock NG dilutions	Prepared sample#	Gonorrhea Panther Measurements [RLU (x1000)]																																	
1.00E+07	3.00E+05	urine	1250	1279	NA	NA	NA	NA	1306	1297	NA	NA	NA	NA	1204	1185	NA	NA	NA	NA	1257	1266	NA	NA	NA	NA	1295	1265	NA	NA	NA	NA	1260	39	10/10 (100%)
		DUS	1243	1206	1269	1291	1273	1266	1151	*	1237	*	1285	1275	1204	1195	1210	1203	1173	1207	1198	*	1217	1207	1191	1240	1249	1223	1225	1255	1254	1260	1230	36	27/27 (100%)
1.00E+06	3.00E+04	urine	1265	1275	NA	NA	NA	NA	1325	1307	NA	NA	NA	NA	1197	1206	NA	NA	NA	NA	1309	1277	NA	NA	NA	NA	1296	1315	NA	NA	NA	NA	1277	44	10/10 (100%)
		DUS	1290	1288	1269	1266	1247	1263	1172	1195	1237	1236	1261	1259	1186	1206	1180	1189	1167	1183	1185	1188	1203	1205	1233	1248	1239	1244	1255	1244	1276	1259	1229	37	30/30 (100%)
1.00E+05	3.00E+03	urine	1259	1288	NA	NA	NA	NA	1300	1300	NA	NA	NA	NA	1159	1177	NA	NA	NA	NA	1249	1268	NA	NA	NA	NA	1282	1311	NA	NA	NA	NA	1259	52	10/10 (100%)
		DUS	1253	1210	1264	1235	1252	1245	1199	1173	1220	1176	1260	1255	1175	1181	1183	1171	1191	1195	1177	1190	1218	1216	1236	1215	1232	1213	1210	1226	1257	1250	1216	30	30/30 (100%)
1.00E+04	3.00E+02	urine	1217	1215	NA	NA	NA	NA	1272	1283	NA	NA	NA	NA	1178	1158	NA	NA	NA	NA	1236	1221	NA	NA	NA	NA	1294	1251	NA	NA	NA	NA	1233	44	10/10 (100%)
		DUS	1190	1190	1137	1139	1176	1164	1118	1128	1146	1087	1181	1207	1104	1117	1057	1058	1136	1117	1135	1121	1092	1107	1165	1186	1169	1127	1091	1090	1109	1146	1133	39	30/30 (100%)
1.00E+03	3.00E+01	urine	1119	1166	NA	NA	NA	NA	1174	1177	NA	NA	NA	NA	1090	1105	NA	NA	NA	NA	1081	1149	NA	NA	NA	NA	1208	1192	NA	NA	NA	NA	1146	45	10/10 (100%)
		DUS	969	934	731	717	699	576	852	*	867	777	816	627	769	749	521	577	971	940	934	929	906	866	1065	1080	849	849	874	856	759	776	822	138	29/29 (100%)
1.00E+02	3.00E+00	urine	847	736	NA	NA	NA	NA	674	763	NA	NA	NA	NA	500	718	NA	NA	NA	NA	807	828	NA	NA	NA	NA	579	672	NA	NA	NA	NA	712	110	10/10 (100%)
		DUS	9	18	9	9	38	20	5	6	8	7	301	277	457	379	469	348	15	10	464	363	447	436	363	374	20	15	410	397	567	473	224	207	16/30 (53%)
1.00E+01	3.00E-01	urine	8	7	NA	NA	NA	NA	7	11	NA	NA	NA	NA	9	10	NA	NA	NA	NA	11	9	NA	NA	NA	NA	12	10	NA	NA	NA	NA	9	2	0/10 (0%)
		DUS	10	10	13	11	7	14	6	*	7	9	31	10	12	13	15	10	9	8	12	10	7	15	10	10	11	10	13	14	8	8	11	5	0/29 (0%)
1.00E+00	3.00E-02	urine	9	9	NA	NA	NA	NA	8	7	NA	NA	NA	NA	11	7	NA	NA	NA	NA	9	10	NA	NA	NA	NA	9	9	NA	NA	NA	NA	9	1	0/10 (0%)
		DUS	11	11	12	9	9	16	9	*	7	7	15	8	7	11	10	10	10	10	8	12	11	10	10	10	9	12	13	10	13	10	10	2	0/29 (0%)

RLU = Relative light unit; SD = standard deviation; Avg = average; NA = not applicable

Panther NG positive = >150 Total RLU (x1000) ; Panther Equivocal region NG only = 60 to <150 Total RLU (x1000) ; Panther Equivocal region CT/GC dual = 85 to <250 Total RLU (x1000)

* = instrument error RDFS/VVFS; invalid result (RDFS; Sample dispense verification failure/ VVFS; Volume verification failure)

#Concentration resulting from addition of 120µl of bacterial stock dilution to 4ml of urine used to prepare DUS and urine samples for testing.

Supplemental Table 4. Aptima Combo 2® Results for 3 specimens excluded from the analysis.

	HQ Aptima Combo 2® Result (Urine) **	NML Aptima Combo 2® Result (Urine)	NML Aptima Combo 2® Result (DUS-A) [RLU(x1000)]	NML Aptima Combo 2® Result (DUS-B) [RLU(x1000)]	NML Aptima Combo 2® Result (DUS-C) [RLU(x1000)]
**Sample 1**	CT positive	CT negative	CT positive [227]	CT negative	CT negative
**Sample 2**	CT positive	CT negative	CT positive [274]	CT negative	CT equivocal [45]
**Sample 3**	NG positive	NG negative	NG negative	NG negative	NG negative

Panther Equivocal region CT only = 25 to <100 Total RLU (x1000) ; NG only = 60 to <150 Total RLU (x1000) ; Panther Equivocal region CT/GC dual = 85 to <250 Total RLU (x1000)

**RLU (x1000) values for HQ tested urine samples were not available

DUS-A = Dried overnight in BSC before packaging

DUS-B = Dried in bag with 2 desiccants

DUS-C = 3-week stability sample, DUS dried in BSC overnight before storage in bag with 1 desiccant

Supplemental Table 5. Diagnostic accuracy of the A-226 DUS in reference to urine for detection of CT in clinical samples obtained from HQ Clinic in Toronto, tested by Aptima Combo 2® Assay.

Urine vs. DUS-A	DUS equivocal considered negative	DUS equivocal considered positive
**True positive**	47	48
**False positive**	0	0
**True negative**	109	109
**False negative**	1	0
**Sensitivity (%, 95% CI)**	97.92 (89.10 - 99.63)	100.00 (92.59 – 100.00)
**Specificity (%, 95% CI)**	100.00 (96.60 – 100.00)	100.00 (96.60 – 100.00)
**PPV (%, 95% CI)**	100.00 (92.44 – 100.00)	100.00 (92.59 – 100.00)
**NPV (%, 95% CI)**	99.09 (95.03 - 99.84)	100.00 (96.63 – 100.00)
**Cohens Kappa(95% CI)**	0.985 (0.955 - 1.000)	1.000 (1.000 -1.000)

Urine vs. DUS-B	DUS equivocal considered negative	DUS equivocal considered positive
**True positive**	46	47
**False positive**	0	0
**True negative**	109	109
**False negative**	2	1
**Sensitivity (%, 95% CI)**	95.83 (86.02 – 98.85)	97.92 (89.10 – 99.63)
**Specificity (%, 95% CI)**	100.00 (96.60 - 100.00)	100.00 (96.60 – 100.00)
**PPV (%, 95% CI)**	100.00 (92.29 - 100.00)	100.00 (92.44 – 100.00)
**NPV (%, 95% CI)**	98.20 (93.67 - 99.50)	99009 (95.03 – 99.84)
**Cohens Kappa(95% CI)**	0.970 (0.928 - 1.000)	0.985 (0.955 – 1.000)

Urine vs. DUS-C	DUS equivocal considered negative	DUS equivocal considered positive
**True positive**	45	46
**False positive**	0	0
**True negative**	109	109
**False negative**	3	2
**Sensitivity (%, 95% CI)**	93.75 (83.16 – 97.85)	95.83 (86.02 – 98.85)
**Specificity (%, 95% CI)**	100.00 (96.60 – 100.00)	100.00 (96.60 – 100.00)
**PPV (%, 95% CI)**	100.00 (92.13 – 100.00)	100.00 (92.29 – 100.00)
**NPV (%, 95% CI)**	97.32 (92.42 – 99.08)	98.20 (93.67 – 99.50)
**Cohens Kappa(95% CI)**	0.954 (0.903 – 1.000)	0.967 (0.928 – 1.000)

Panther Equivocal region CT only = 25 to <100 Total RLU (x1000) ; Panther Equivocal region CT/GC dual = 85 to <250 Total RLU (x1000)

DUS-A = Dried overnight in BSC before packaging

DUS-B = Dried in bag with 2 desiccants

DUS-C = 3-week stability sample, DUS dried in BSC overnight before storage in bag with 1 desiccant

Supplemental Table 6. Diagnostic accuracy of the A-226 DUS in reference to urine for detection of NG in clinical samples obtained from HQ Clinic in Toronto, tested by Aptima Combo 2® Assay.

Urine vs. DUS-A	DUS equivocal considered negative	DUS equivocal considered positive
**True positive**	51	52
**False positive**	1	1
**True negative**	102	102
**False negative**	3	2
**Sensitivity (%, 95% CI)**	94.44 (84.89 – 99.83)	96.30 (87.46 – 98.98)
**Specificity (%, 95% CI)**	99.03 (94.70 - 99.83)	99.03 (94.70 – 99.83)
**PPV (%, 95% CI)**	98.08 (89.88 – 99.66)	98.11 (90.06 – 99.67)
**NPV (%, 95% CI)**	97.14 (91.93 - 99.02)	98.08 (93.26 - 99.47)
**Cohens Kappa(95% CI)**	0.943 (0.888 - 0.998)	0.958 (0.910 – 1.000)

Urine vs. DUS-B	DUS equivocal considered negative	DUS equivocal considered positive
**True positive**	50	52
**False positive**	0	0
**True negative**	103	103
**False negative**	4	2
**Sensitivity (%, 95% CI)**	92.59 (82.45 – 97.08)	96.30 (87.46 – 98.98)
**Specificity (%, 95% CI)**	100.00 (96.40 – 100.00)	100.00 (96.40 – 100.00)
**PPV (%, 95% CI)**	100.00 (92.87 – 100.00)	100.00 (93.12 – 100.00)
**NPV (%, 95% CI)**	96.26 (90.78 – 98.54)	98.10 (93.32 – 99.49)
**Cohens Kappa(95% CI)**	0.943 (0.887 – 0.998)	0.972 (0.932 – 1.000)

Urine vs. DUS-C	DUS equivocal considered negative	DUS equivocal considered positive
**True positive**	52	53
**False positive**	0	0
**True negative**	103	103
**False negative**	2	1
**Sensitivity (%, 95% CI)**	96.30 (87.46 – 98.98)	98.15 (90.23 – 99.67)
**Specificity (%, 95% CI)**	100.00 (96.40 – 100.00)	100.00 (96.40 -100.00)
**PPV (%, 95% CI)**	100.00 (93.12 – 100.00)	100.00 (93.24 -100.00)
**NPV (%, 95% CI)**	98.10 (93.32 – 99.48)	99.04 (94.75 – 99.83)
**Cohens Kappa(95% CI)**	0.972 (0.932 – 1.000)	0.986 (0.958 – 1.000)

Panther Equivocal region NG only = 60 to <150 Total RLU (x1000) ; Panther Equivocal region CT/NG dual = 85 to <250 Total RLU (x1000)

DUS-A = Dried overnight in BSC before packaging

DUS-B = Dried in bag with 2 desiccants

DUS-C = 3-week stability sample, DUS dried in BSC overnight before storage in bag with 1 desiccant

Supplemental Table 7. Diagnostic accuracy of the A-226 DUS in reference to urine for detection of CT and NG in clinical samples obtained from BCCDC, tested by Aptima Combo 2® Assay.

Urine vs. DUS	Chlamydia (CT)
**True positive**	32
**False positive**	0
**True negative**	71
**False negative**	1
**Sensitivity (%, 95% CI)**	96.97 (84.68 – 99.46)
**Specificity (%, 95% CI)**	100.00 (94.87 – 100.00)
**PPV (%, 95% CI)**	100.00 (89.28 – 100.00)
**NPV (%, 95% CI)**	98.61 (92.54 – 99.75)
**Cohens Kappa(95% CI)**	0.98 (0.93-1.00)

Urine vs. DUS-A	Gonorrhea (NG)
**True positive**	12
**False positive**	1
**True negative**	91
**False negative**	0
**Sensitivity (%, 95% CI)**	100.0 (75.75 – 100.0)
**Specificity (%, 95% CI)**	98.91 (94.10 – 99.81)
**PPV (%, 95% CI)**	92.31 (66.69 – 98.63)
**NPV (%, 95% CI)**	100.00 (95.95 – 100.00)
**Cohens Kappa(95% CI)**	0.955 (0.866 – 1.000)

Urine was tested by BCCDC using Aptima assay.
DUS were prepared at BCCDC (DUS-A protocol) and shipped to NML for elution and testing.
No equivocal results were obtained from DUS testing or reported from BCCDC urine testing.

Supplemental Table 8. Diagnostic accuracy of the A-226 DUS in reference to urine for detection of CT in clinical samples obtained from HQ Clinic in Toronto, tested by CT RT-PCR (CT Cryptic plasmid target).

Urine vs. DUS-A	
True positive	45
False positive	2
True negative	107
False negative	3
Sensitivity (%, 95% CI)	93.75 (83.16 - 97.85)
Specificity (%, 95% CI)	98.17 (93.56 - 99.50)
PPV (%, 95% CI)	95.74 (85.75 - 98.83)
NPV (%, 95% CI)	97.27 (92.48 - 99.07)
Cohens Kappa(95% CI)	0.925 (0.860 - 0.990)
**Urine vs. DUS-B**	
True positive	44
False positive	1
True negative	108
False negative	4
Sensitivity (%, 95% CI)	91.67 (80.45 – 96.71)
Specificity (%, 95% CI)	99.08 (94.99 – 99.84)
PPV (%, 95% CI)	97.78 (88.43 – 99.61)
NPV (%, 95% CI)	96.43 (91.18 – 98.60)
Cohens Kappa(95% CI)	0.924 (0.858 – 0.990)
**Urine vs. DUS-C**	
True positive	45
False positive	0
True negative	109
False negative	3
Sensitivity (%, 95% CI)	93.75 (83.16 – 97.85)
Specificity (%, 95% CI)	100.00 (96.60 – 100.00)
PPV (%, 95% CI)	100.00 (92.13 – 100.00)
NPV (%, 95% CI)	97.32 (92.42 – 99.08)
Cohens Kappa(95% CI)	0.954 (0.903 – 1.000)

DUS-A = Dried overnight in BSC before packaging
DUS-B = Dried in bag with 2 desiccants
DUS-C = 3-week stability sample, DUS dried in BSC overnight before storage in bag with 1 desiccant

Supplemental Table 9. Diagnostic accuracy of the A-226 DUS in reference to urine for detection of NG in clinical samples obtained from HQ Clinic in Toronto, tested by porA RT-PCR (only paired NG positive samples by NML Aptima Combo 2® Assay on urine were tested).

Urine vs. DUS-A[a]	
True positive	54
False negative	0
Sensitivity (%, 95% CI)	100.00 (93.36 – 100.00)
PPV (%, 95% CI)	100.00 (93.36 – 100.00)
Urine vs. DUS-B[b]	
True positive	10
False negative	0
Sensitivity (%, 95% CI)	100.00 (72.25 – 100.00)
PPV (%, 95% CI)	100.00 (72.25 – 100.00)

[a] Only NG positive DUS-A were tested by RT-PCR
[b] Only 10 NG positive DUS-B were tested by RT-PCR
DUS-A = Dried overnight in BSC before packaging
DUS-B = Dried in bag with 2 desiccants

Supplemental Table 10. Chlamydia genotyping results indicating samples that were sequenced and genotype agreement between urine and DUS samples.

	CT genotyping result				CT genotype agreement between sample types		
	**Urine**	**DUS-A**	**DUS-B**	**DUS-C**	**Urine vs. DUS-A**	**Urine vs. DUS-B**	**Urine vs. DUS-C**
**1**	1	1	1	1	1	1	1
**2**	1	1	1	1	1	1	1
**3**	1	1	1	1	1	1	1
**4**	1	1	1	1	1	1	1
**5**	1	1	1	1	1	1	1
**6**	0	0	0	0			
**7**	0	0	0	0			
**8**	1	1	1	1	1	1	1
**9**	0	0	0	0			
**10**	1	1	1	1	1	1	1
**11**	1	1	1	1	1	1	1
**12**	1	1	0	1	1		1
**13**	1	0	0	0			
**14**	1	0	0	0			
**15**	1	1	1	1	1	1	1
**16**	1	1	1	0	1	1	
**17**	1	1	1	1	1	1	1
**18**	1		1	1		1	1
**19**	1	1	1	1	1	1	1
**20**	1	1	0	0	1		
**21**	1	0	0	0			
**22**	1	1	1	1	1	1	1
**23**	1	0	0	0			
**24**	1	1	1	1	1	1	1
**25**	1	1	1	1	1	1	1
**26**	1	1	0	1	1		1
**27**	1	0	0	0			
**28**	1	1	1	1	1	1	1
**29**	1	1	1	1	1	1	1
**30**	1	1	1	1	1	1	1
**31**	1	1	1	1	1	1	1
**32**	0	0					
**33**	1	1	1	1	1	1	1
**34**	1	1	1	0	1	1	
**35**	1	1	1	1	1	1	1
**36**	1	1	1	1	1	1	1
**37**	1	0	0	0			
**38**	1	1	1	1	1	1	1
**39**	1	0	0	0			
**40**	1	1	1	1	1	1	1
**41**	1	0	0	0			
**42**	1	1	1	1	1	1	1
**43**	1	1	0	1	1		1
**44**	1	1	1	1	1	1	1
**45**	0	0		0			
**46**	1	1	1	1	1	1	1
**47**	1	0	0	0			
**48**	0	0					
	**1= Sequenced; 0= Not sequenced**				**1= Genotype agreement; 0= Genotype disagreement**		

= Genotyping was not attempted on sample

= Genotype agreement not available due to lack of sequence data

DUS-A = Dried overnight in BSC before packaging ; DUS-B = Dried in bag with 2 desiccants ; DUS-C = 3-week stability sample, DUS dried in BSC overnight before storage in bag with 1 desiccant

Supplemental Table 11. 2x2 table for sensitivity and specificity calculations for CT sequencing in urine and DUS sample types

		Urine		Sensitivity (%, 95% CI)	Specificity (%, 95% CI)
		Sequence	No Sequence		
**Urine vs. DUS-A**	**Sequence**	32	0	78.05(63.29 – 88.00)	100.00 (60.97 – 100.00)
	**No Sequence**	9	6		
**Urine vs. DUS-B**	**Sequence**	29	0	69.05 (53.97 – 80.93)	100.00 (43.85 – 100.00)
	**No Sequence**	13	3		
**Urine vs. DUS-C**	**Sequence**	30	0	71.43 (56.43 – 82.83)	100.00 (51.01 – 100.00)
	**No Sequence**	12	4		

DUS-A = Dried overnight in BSC before packaging
DUS-B = Dried in bag with 2 desiccants
DUS-C = 3-week stability sample, DUS dried in BSC overnight before storage in bag with 1 desiccant

Supplemental Table 12. Gonorrhea sequence typing results indicating samples that were sequenced and sequence type agreement between urine and DUS samples.

	NG-MAST results			NG-MAST agreement between sample types	
	Urine	DUS-A	DUS-B*	Urine vs. DUS-A	Urine vs. DUS-B
**1**	1	1	1	1	1
**2**	1	1	1	1	1
**3**	1	1		1	
**4**	1	1	0	1	
**5**	1	1	1	1	1
**6**	1	1	1	1	1
**7**	1	1	1	1	1
**8**	1	1	1	1	1
**9**	1	1	1	1	1
**10**	1	1	1	1	1
**11**	1	1	0	1	
**12**	1	1		1	
**13**	1	1		1	
**14**	1	1		1	
**15**	0	1			
**16**	1	1		1	
**17**	1	1		1	
**18**	0	1			
**19**	1	1		1	
**20**	1	1		1	
**21**	1	1		1	
**22**	1	1		1	
**23**	1	1		1	
**24**	1	1		1	
**25**	1	1		1	
**26**	1	1		1	
**27**	1	1		1	
**28**	1	1		1	
**29**	1	1		1	
**30**	1	1		1	
**31**	1	1		1	
**32**	1	1		1	
**33**	0	1			
**34**	1	1		1	
**35**	1	1		1	
**36**	1	1		1	
**37**	0	1			
**38**	1	1		1	
**39**	1	1		1	
**40**	1	1		1	
**41**	1	0			
**42**	1	1		1	
**43**	1	1		1	
**44**	1	1		1	
**45**	1	1		1	
**46**	1	1		1	
**47**	1	1		1	
**48**	1	1		1	
**49**	1	1		1	
**50**	1	0			
**51**	1	1		1	
**52**	1	1		1	
**53**	1	1		1	
**54**	1	1		1	
	1= Typeable; 0= Non-typable			1= Type agreement; 0= Type disagreement	

= NG-MAST was not attempted on sample
= Sequence type agreement not available due to lack of sequence data
DUS-A = Dried overnight in BSC before packaging ; DUS-B = Dried in bag with 2 desiccants
*Note: only 10 DUS-B were typed by NG-MAST

Supplemental Table 13. 2x2 table for sensitivity calculations for NG sequencing in urine and DUS sample types

		Urine		Sensitivity (%, 95% CI)
		**Sequence**	**No Sequence**	
**Urine vs. DUS-A**	**Sequence**	48	4	96.00(86.54 – 98.90)
	**No Sequence**	2	0	
**Urine vs. DUS-B***	**Sequence**	8	0	80.00 (49.02 – 94.33)
	**No Sequence**	2	0	

DUS-A = Dried overnight in BSC before packaging
DUS-B = Dried in bag with 2 desiccants
*Note: only 10 DUS-B were typed by NG-MAST

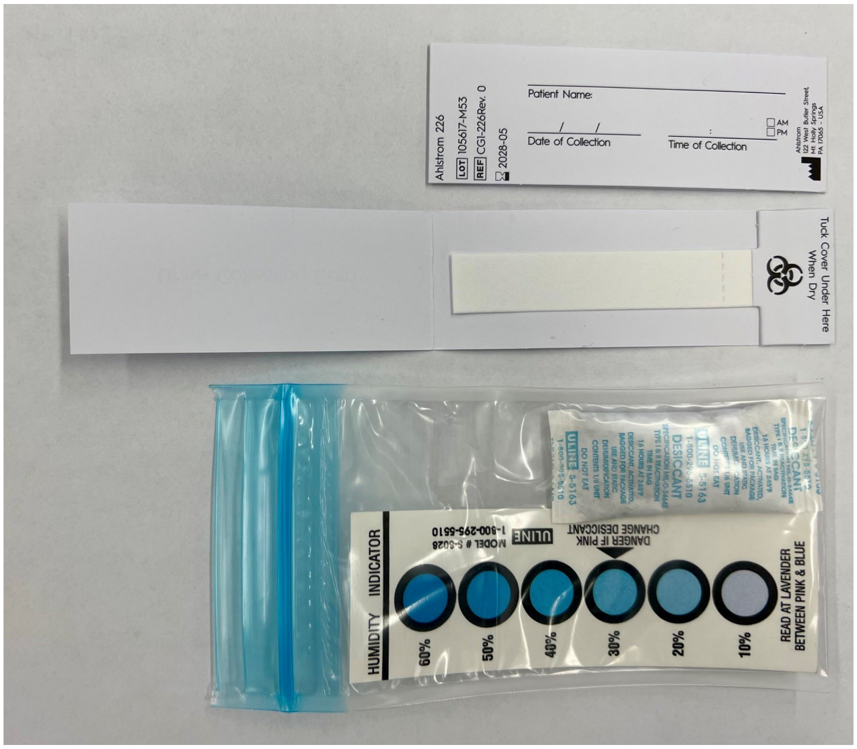

Supplemental Figure 1. DUS storage. Prepared DUS were placed in the storage bag (Uline, product S-17703) containing a clay desiccant and a humidity indicator card (Uline, product S-5163 and S-8028).

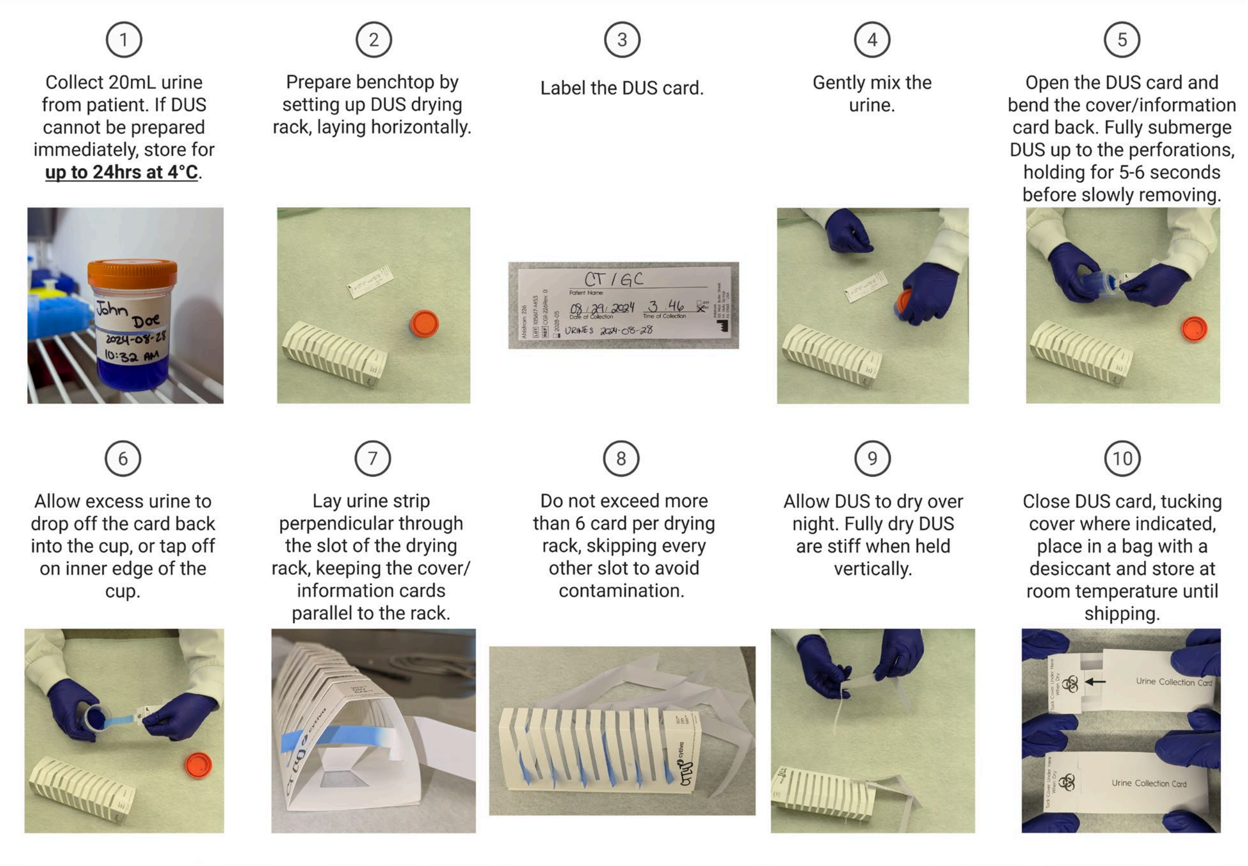

Supplemental Figure 2. DUS preparation. A urine collection cup/container, containing first void urine (~20 mL), was swirled to ensure the mixing of the sample. The filter paper strip was submerged into urine up to the perforations for approximately 5 seconds. The strip was removed from the cup and placed on a drying rack. The strips were sufficiently spaced to ensure no cross contamination and left to dry at room temperature (22ºC) overnight in a biological safety cabinet. The next morning, the strips were placed in the storage bag containing a clay desiccant and a humidity indicator card. Created in BioRender. (2025) https://BioRender.com/b1hwjqd

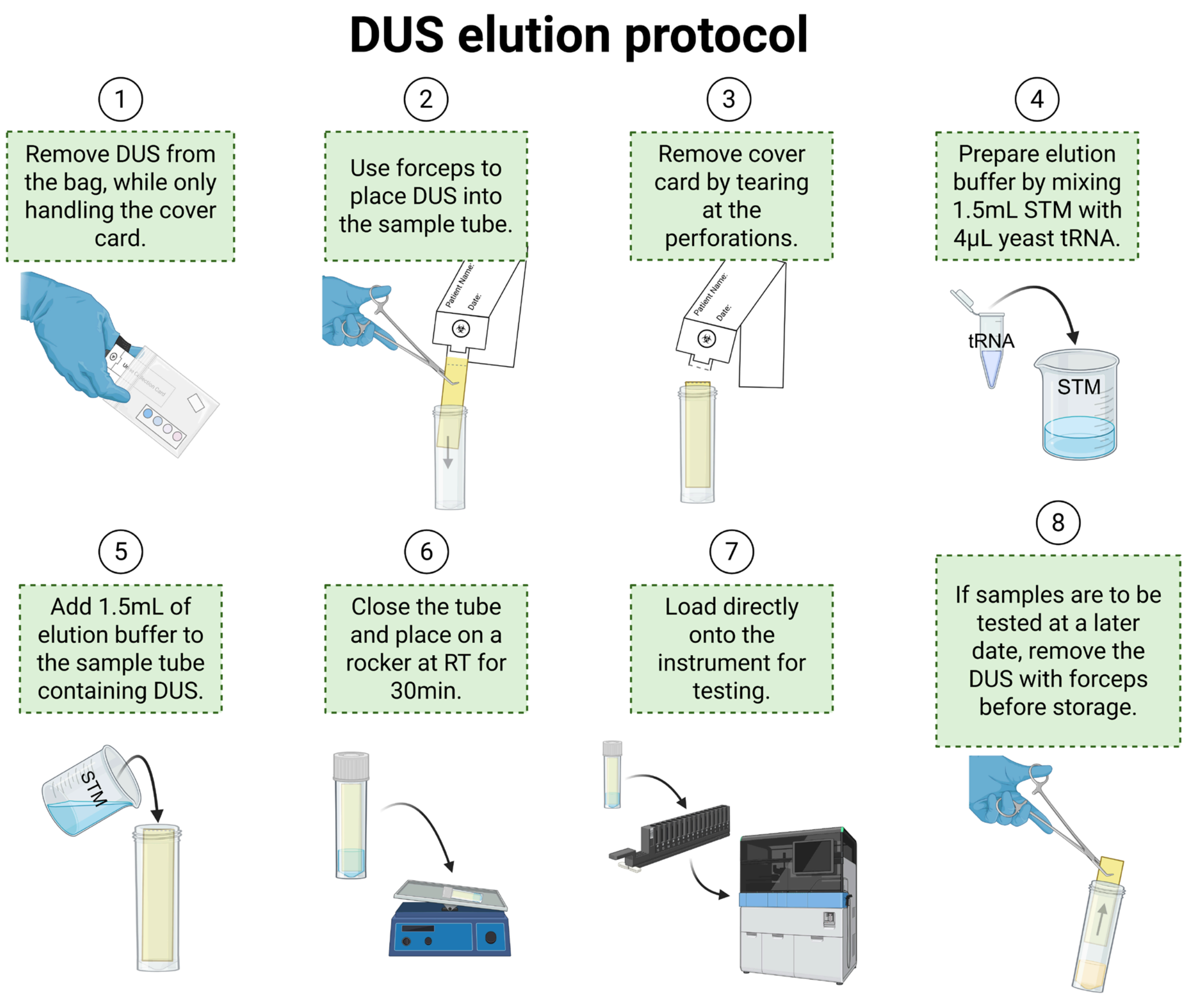

Supplemental Figure 3. DUS elution. Prior to testing, DUS filter papers were placed into Aptima specimen collection tubes (Hologic, product FAB-18184) using clean forceps. Strips were sized to fit snugly along the wall of the Aptima tube when capped with penetrable caps (Hologic, product #105668). Sample elution was done by adding 1.5 mL of Specimen Transport Medium (STM) (Hologic, PRD-04423) spiked with 4µL of yeast tRNA (Invitrogen, product # AM7119). Samples were left at room temperature for 30 minutes on a nutating mixer at 30 rpm. Samples that were tested immediately were loaded directly onto the Hologic Panther system and tested with the Aptima Combo 2® Kit (Hologic, product PRD-05571/PRD-05576) without removal of the strip. For long term sample storage, the filter paper was removed with tweezers and discarded in order to prevent paper degradation. The DUS elution volume was based on the need to have sufficient sample for duplicate testing for the Aptima Combo 2® Assay and/or downstream pathogen characterization. Created in BioRender. (2025) https://BioRender.com/b1hwjqd

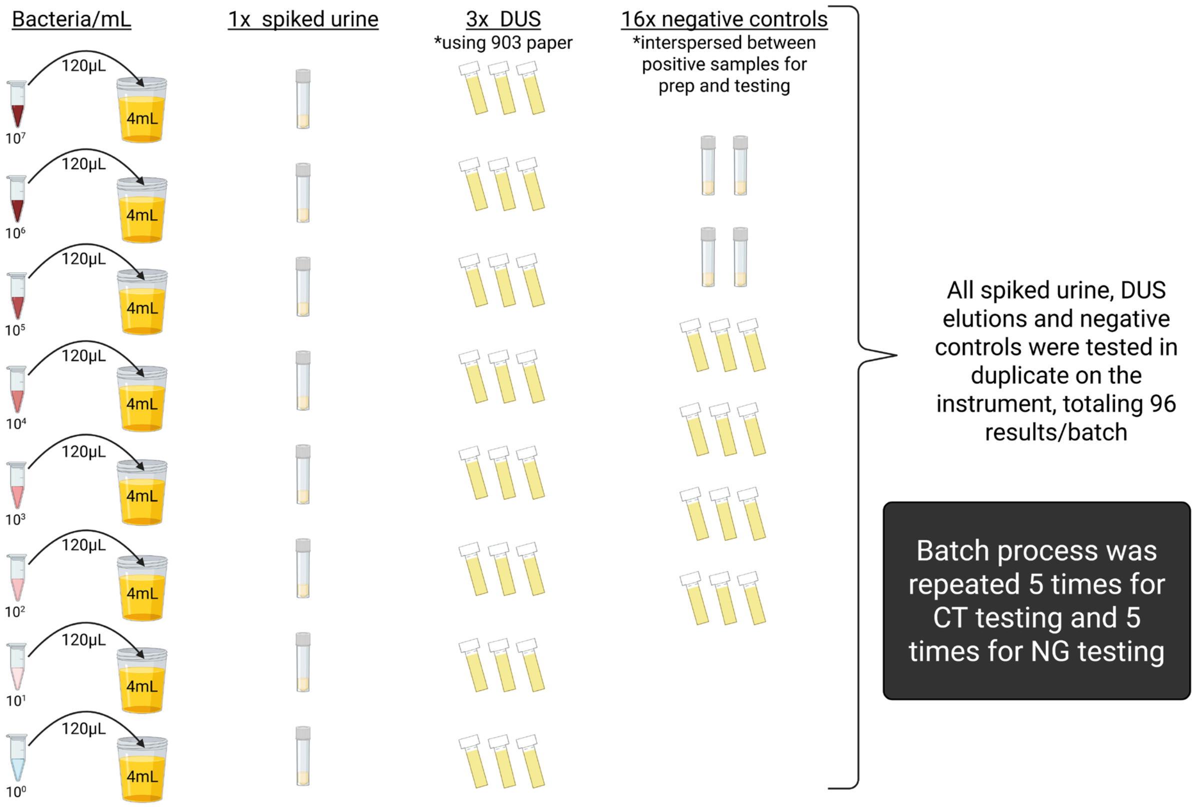

Supplemental Figure 4. Diagram of the DUS evaluation using the prepared CT and NG bacterial stock dilutions. For each bacterial concentration, spiked urine was prepared by adding 120 µL of bacterial stock to 4 mL of urine. From this, 1 mL of urine and 3 DUS cards were prepared. As negative controls, three 1 mL aliquots of urine and 12 DUS cards were prepared using unspiked urine. Each sample was tested in duplicate using the Aptima Combo 2® Assay, and the entire procedure was repeated five times. Experiments were performed using the Whatman™ 903 filter strip. Created in BioRender. (2025) https://BioRender.com/b1hwjqd

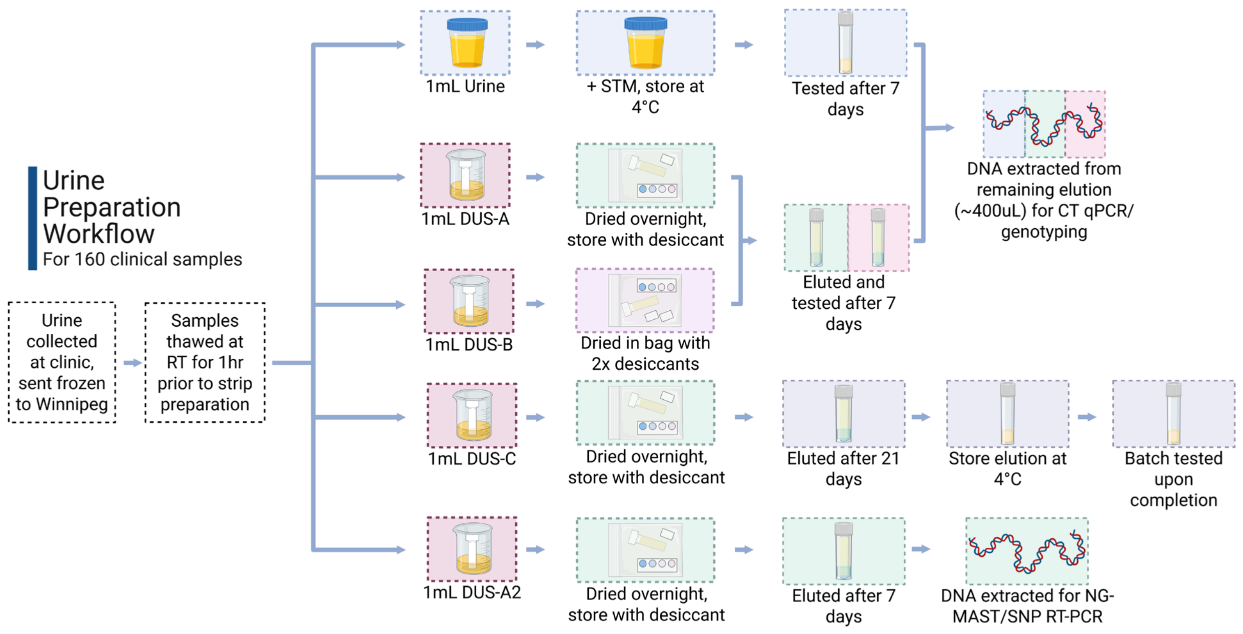

Supplemental Figure 5. Study diagram for the DUS clinical validation using specimens obtained from the HQ Clinic in Toronto. For urine testing, a 1 mL aliquot of urine was transferred into an Aptima specimen collection tube with 1 mL of specimen transport medium. Aliquoted urine was stored at 4°C and tested the same day the following week. Additionally, 4 DUS strips were prepared for each sample. Two strips were prepared as per the original protocol, dried overnight and stored for one week prior to elution and testing (DUS-A and DUS-A2). DUS-A was eluted and tested using the Aptima Combo 2® Assay on the same day the following week. DUS-A2 was eluted and DNA was extracted using the MP96 instrument for downstream in-house developed NG testing. To assess a more patient-friendly approach to sample preparation, DUS-B was placed directly into the storage bag with two desiccants, bypassing the overnight drying step. The sample was eluted and tested using the Aptima Combo 2® Assay the same day the following week. Finally, DUS-C was prepared, dried overnight, and stored for 3 weeks prior to elution to assess long-term sample stability. Once eluted, DUS-C samples were stored at 4°C and batch tested after all samples reached the 3-week storage mark. For both DUS-A and DUS-B, DNA was extracted using the MP96 system from the residual sample remaining after Aptima Combo 2® assay testing. Extracted DNA was used for the CT testing via the in-house developed RT-PCR assay and downstream CT genotyping and NG-MAST characterization. A-226 filter paper DUS was used for this study. Created in BioRender. (2025) https://BioRender.com/b1hwjqd

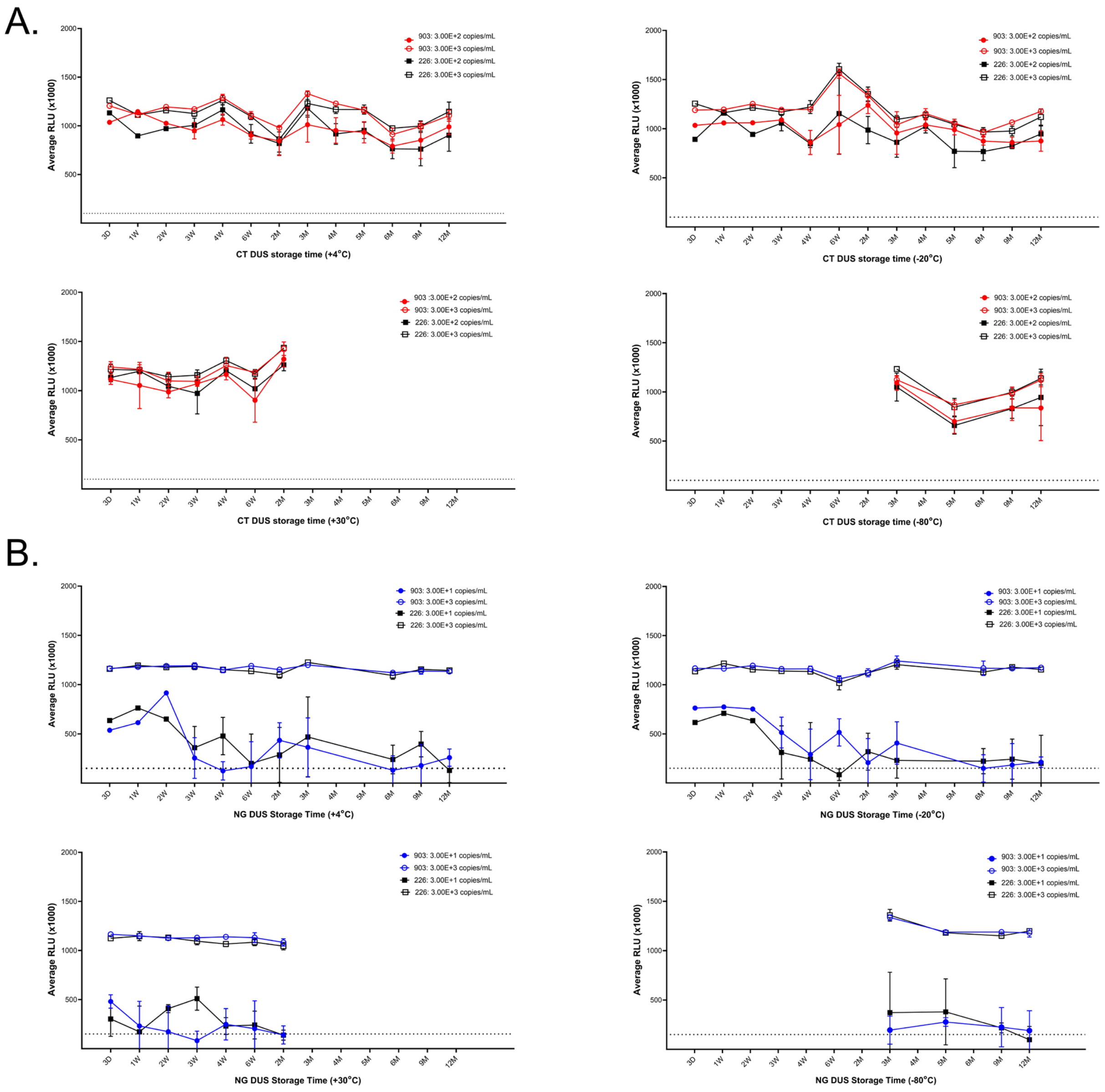

Supplemental Figure 6. DUS stability at +4ºC, -20ºC, +30ºC and, -80ºC for **A**. CT and **B**. NG over 12 month period. DUS stability at +30ºC was only monitored until 2 months of storage and -80ºC was only monitored following 3 months of storage. Aptima Combo 2® Assay RLU (x1000) values [with mean and standard deviation (SD)] are indicated for samples collected on Whatman™ 903 (circle) and A-226 (square) filter papers at 3.00E+3 copies/ml for both CT and NG (unfilled circles and squares), and 3.00E+2 for CT and 3.00E+1 for NG (filled circles and squares). D = day, W = week, M = month. Dashed line indicates positive RLU (x1000) threshold (CT = 100; NG = 150).

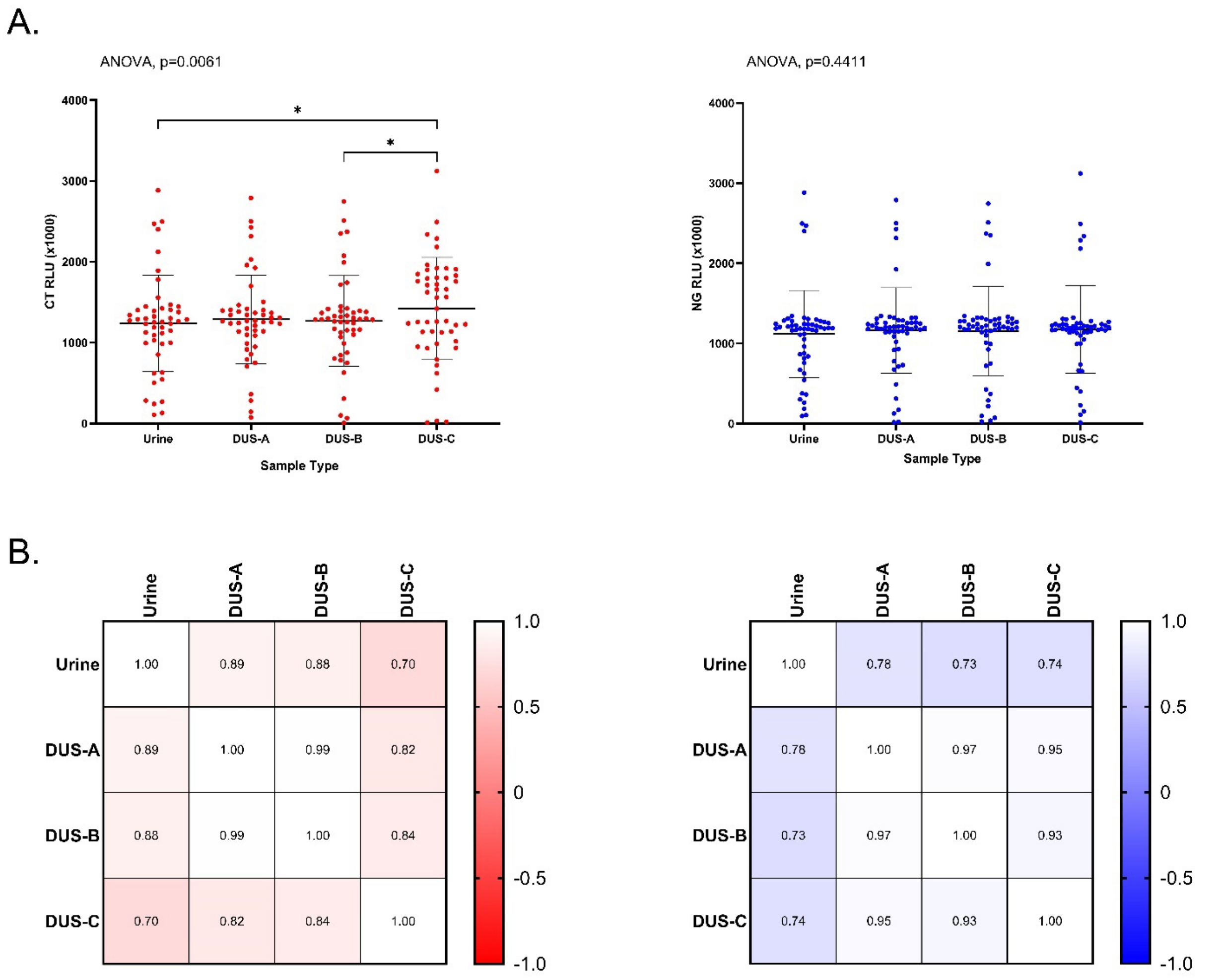

Supplemental Figure 7. A. RLU (x1000) values for CT (red) and NG (blue) for different sample collection methods and conditions. ANOVA with Tukey's post hoc test with significant values indicated with * p<0.05. Lines indicate mean and SD. **B**. Heatmap of correlations between RLU values obtained for different sample types (CT in red and NG in blue). Pearsons correlation r value is indicated in the heat map with all p values falling below p< 0.0001. Sample types: urine, DUS-A (A-226 DUS dried overnight prior to storage), DUS-B (A-226 DUS placed directly into the sample collection bag with 2 desiccants), DUS-C (A-226 DUS dried overnight and stored at RT for 3 weeks prior to elution).

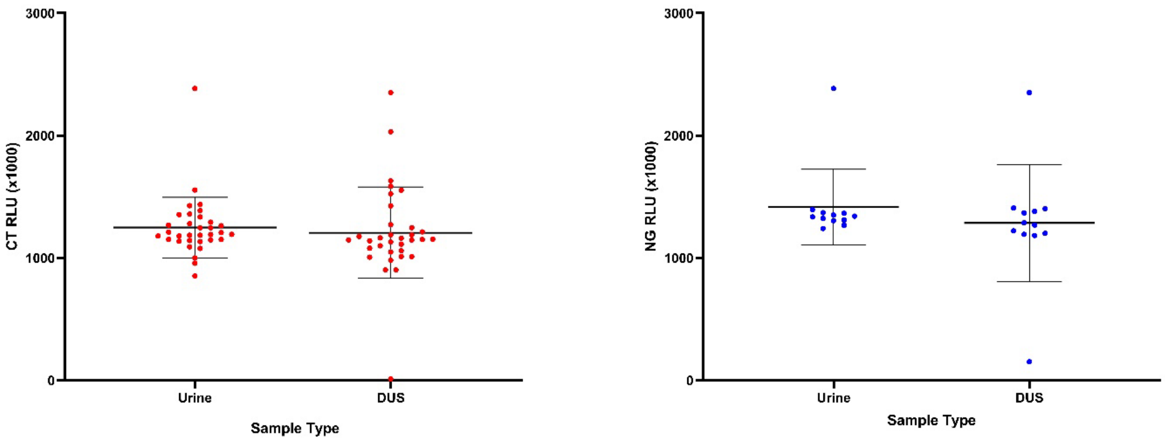

Supplemental Figure 8. BCCDC pilot study RLU (x1000) values for urine and DUS for CT (red, n=33) and NG (blue, n= 11). Lines indicate mean and SD.

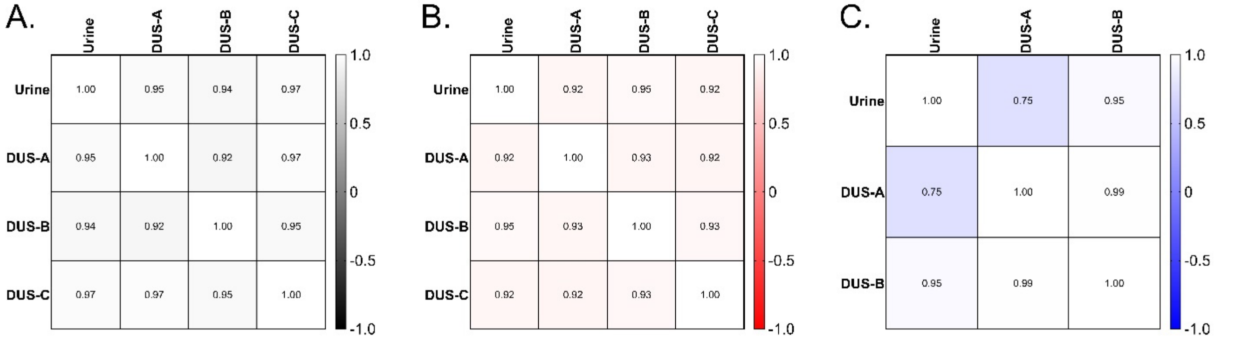

Supplemental Figure 9. Heatmap of RT-PCR Cq correlations for RNAseP (**A**) , CT Cryptic plasmid (**B**) and RT-PCR NG correlations for *porA* targets (**C**) between different sample types. Pearson correlation coefficient is indicated in the heatmap with all p values falling below 0.0001. Sample types: urine, DUS-A (A-226 DUS dried overnight prior to storage), DUS-B (A-226 DUS placed directly into the sample collection bag with 2 desiccants), DUS-C (A-226 DUS dried overnight and stored at RT for 3 weeks prior to elution).
